# Toll-like receptor-2 induced inflammation causes local bone formation and activates canonical Wnt signaling

**DOI:** 10.3389/fimmu.2024.1383113

**Published:** 2024-04-05

**Authors:** Petra Henning, Ali Kassem, Anna Westerlund, Pernilla Lundberg, Cecilia Engdahl, Vikte Lionikaite, Pernilla Wikström, Jianyao Wu, Lei Li, Catharina Lindholm, Pedro P. C. de Souza, Sofia Movérare-Skrtic, Ulf H. Lerner

**Affiliations:** ^1^ Sahlgrenska Osteoporosis Centre, Centre for Bone and Arthritis Research, Department of Internal Medicine and Clinical Nutrition, Institute for Medicine, Sahlgrenska Academy at University of Gothenburg, Gothenburg, Sweden; ^2^ Department of Molecular Periodontology, Umeå University, Umeå, Sweden; ^3^ Department of Rheumatology and Inflammation Research, Institute for Medicine, Sahlgrenska Academy at University of Gothenburg, Gothenburg, Sweden; ^4^ Department of Medical Biosciences, Section of Pathology, Umeå University, Umeå, Sweden; ^5^ Innovation in Biomaterials Laboratory, Federal University of Goiás, Goiania, Brazil

**Keywords:** toll-like receptors, osteoclasts, osteoblasts, bone formation, Wnt signaling

## Abstract

It is well established that inflammatory processes in the vicinity of bone often induce osteoclast formation and bone resorption. Effects of inflammatory processes on bone formation are less studied. Therefore, we investigated the effect of locally induced inflammation on bone formation. Toll-like receptor (TLR) 2 agonists LPS from *Porphyromonas gingivalis* and PAM2 were injected once subcutaneously above mouse calvarial bones. After five days, both agonists induced bone formation mainly at endocranial surfaces. The injection resulted in progressively increased calvarial thickness during 21 days. Excessive new bone formation was mainly observed separated from bone resorption cavities. Anti-RANKL did not affect the increase of bone formation. Inflammation caused increased bone formation rate due to increased mineralizing surfaces as assessed by dynamic histomorphometry. In areas close to new bone formation, an abundance of proliferating cells was observed as well as cells robustly stained for Runx2 and alkaline phosphatase. PAM2 increased the mRNA expression of *Lrp5*, *Lrp6* and *Wnt7b*, and decreased the expression of *Sost* and *Dkk1*. *In situ* hybridization demonstrated decreased *Sost* mRNA expression in osteocytes present in old bone. An abundance of cells expressed *Wnt7b* in Runx2-positive osteoblasts and ß-catenin in areas with new bone formation. These data demonstrate that inflammation, not only induces osteoclastogenesis, but also locally activates canonical WNT signaling and stimulates new bone formation independent on bone resorption.

## Introduction

Local bone loss is a common consequence of inflammatory processes in vicinity of the skeleton. Articular bone erosion and breakdown of juxtaarticular bone in patients with rheumatoid arthritis (RA) or psoriasis arthritis, and of alveolar bone surrounding roots of teeth in patients with periodontitis, are important events causing joint destruction and tooth loss (1–4). Inflammation induced bone loss is also the reason why joint prostheses and tooth implants can lose their attachments to bone (5, 6). Bone is lost adjacent to inflammatory processes mainly due to increased osteoclast formation and excessive bone resorption (7–11). In contrast to physiological remodeling of bone, where bone resorption is coupled to increased bone formation in order to keep bone mass constant (12), inflammation induced bone resorption is commonly associated with decreased bone formation which further contributes to decreased bone mass (2).

Evidence that inflammation inhibits bone formation has been obtained in several studies including in a mouse model with K/Bx/N serum transfer arthritis (13). Bone surfaces with active mineralization were reduced in areas adjacent to inflammation compared to surfaces with no inflammation. Although an abundance of early osteoblast progenitors expressing Runx2 was observed in the vicinity of inflammation, the numbers of cells expressing markers of late osteoblasts such as alkaline phosphatase (ALP) and osteocalcin were reduced. Inflammation induced decrease of bone formation has also been observed in collagen-induced arthritis, in human tumor necrosis factor (TNF) transgenic (hTNFtg) mice and in adjuvant arthritic rats (14–16). In these arthritic models, inhibition of bone formation has been attributed to TNF and increased expression of the WNT inhibitor DKK1. Further support for the view that TNF may be important for inhibition of bone formation in inflammatory conditions comes from *in vitro* studies using cell lines, and primary human and mouse cells (rev. in (17)), showing that TNF can decrease osteoblast differentiation and synthesis of bone matrix proteins (18) through enhanced degradation of the osteoblastic transcription factor Runx-2 (19). In contrast to these findings, however, there are several reports (rev. in (17)) showing that TNF can stimulate osteoblastic differentiation using human, rat and mouse mesenchymal stem cells through upregulation of the expression of *Runx2* and *Sp7* (encoding osterix) (20, 21). TNF has also been reported to enhance the expression of *Wnt5a* and *Wnt10b* in human mesenchymal stem cells, in which TNF-stimulation causes increased ALP and enhanced mineralization (22). Similar to these paradoxical findings, it has been observed that bone formation can either be stimulated (20) or inhibited (23) by activation of NF-κB, an important transcription factor involved in TNF signaling.

Enhanced numbers of osteoblasts and osteoid deposition at endosteal surfaces have been observed in patients with RA (24). Regensburger et al. reported that patients with RA have increased areas with osteosclerotic, endocortical bone in the joints in addition to the osteolytic lesions (25). More frequently, new bone formation in addition to erosions has been observed in the joints of patients with psoriatic arthritis (26, 27). Ankylosing spondylitis is another inflammatory disease characterized by bone loss, increased risk for osteoporosis and new local bone formation called syndesmophytes (28). Osteoarthritis is an inflammatory disease affecting joints in which new bone formation can be observed in the form of enhanced subchondral bone mass and sometimes as osteophytes (29). These findings indicate that not only bone erosions can be observed in patients with arthritic diseases, but that also that new bone formation can be induced in certain areas in joints harboring inflammatory processes.

Loss of bone due to inflammatory induced increased bone resorption is the cause of loosened joint prosthesis, however, enhanced bone formation can also be observed in these patients, as demonstrated by increased, local uptake of technetium (10). Histopathological analysis has shown the presence of numerous osteoclasts as well as distinct areas with cuboid, active osteoblasts and new bone formation (30). Similarly, enhanced bone formation, as indicated by increased technetium uptake, has been observed in the jaw bones surrounding teeth with active periodontitis (31). In patients with chronic osteomyelitis and Garre´s osteomyelitis, bone marrow inflammation and bone loss are seen together with osteosclerotic areas (32, 33).

The observations in humans indicate that although increased bone resorption is a common consequence of inflammatory process within or in the vicinity of the skeleton, there is also evidence of new bone formation in response to inflammation. Thus, the skeleton can respond to inflammatory processes similar to the response to skeletal metastases of malignant tumors where both osteolytic and osteosclerotic reactions can be observed (34).

Experimental evidence that increased osteoblast activity and bone formation can be induced close to inflammatory sites has been provided by observations in hTNFtg mice. Overexpression of hTNF results not only in joint inflammation, osteoclast formation and periarticular bone loss but also in enhanced bone formation (16, 35). In these studies, the numbers of osteoblasts expressing osteocalcin were enhanced at endosteal surfaces close to inflammatory infiltrates. Increased osteoid formation and enhanced mineralizing surfaces were also observed in these areas. In addition, new bone formation adjacent to bone resorption has been observed 2-4 weeks after injection of *Staphylococcus aureus* in mouse tibia (36).

Local injection of bacterial components on the top of calvaria is an experimental model that has been used to stimulate inflammation-induced osteoclast formation and to assess effects of potentially pro- and anti-osteoclastogenic molecules by us (37, 38) and several other groups since the pioneering report by Boyce et al. (39). In the present study, we investigated if local inflammation in the periosteum of mouse calvaria induced by Toll-like receptor 2 (TLR2) agonists is associated with effects on bone formation. We, here, show that periosteal inflammation not only induces local and systemic osteoclast formation and bone resorption, but also local, excessive new bone formation independent on bone resorption and associated with increased WNT signaling.

## Materials and methods

### Animals

Mice were either CsA from our own inbred colony, *wild type* C57BL/6J or *Tlr2-*deficient*^-^* (B6.129 Tlr^2tm1Kir^/J) mice from Jackson laboratories at Umeå University, or C57BL/6N mice from Charles River Laboratories at University of Gothenburg. Results obtained with the different *wild type* mouse strains were comparable in all experiments. Ethical permit for the use of mice was approved by the Ethical Committee for Animal Research in Gothenburg and Umeå and the care of the animals was in accordance with relevant guidelines and regulations. Mice were randomized into body weight matched groups and treatment groups were housed in separate cages. All treatment groups included 10 mice at the start of the experiment. Mice that showed severe signs of systemic response and lost more than 20% body weight were anesthetized and sacrificed. At the end of experiments, femur and calvaria were dissected and used for either computed tomography (CT), RNA preparation or different histological analyses.

### Induction of inflammation

Five to seven week-old male mice were injected with 100 µl of either *Porphyromonas gingivalis* LPS (500 µg)(version 10G2D-MY; Invivogen), palmitoyl-2-Cys-Ser-(Lys)_4_ (PAM2; 50 µg)(Invivogen) or vehicle (NaCl, 9 mg/ml) *s.c.* over the calvaria. After 1-21 days, the calvarial bones and femur were dissected and bone mass analyzed with CT and then processed for histological analyses, dynamic histomorphometry or *in situ* hybridization. In separate experiments, mice were similarly treated and calvarial bones harvested for gene expression analysis. The LPS and PAM2 preparations have previously been shown to be without effects on periosteal inflammation and effects on mouse calvaria in *Tlr2*-deficient mice (38).

### Treatment with anti-RANKL antibodies and zoledronic acid

To block osteoclast formation and bone resorption mice were treated with anti-RANKL or zoledronic acid in combination with PAM2. Anti-RANKL (clone IK22/5, Nordic Biosite, 1.5 mg/ml) or vehicle (NaCl, 9 mg/ml) were given *i.p* (200 µl) three days before PAM2 (day -3), at the same day as PAM2 (day 0) and three days after PAM2 injection (day 3). Zoledronic Acid (Actavis, Teva, 20 µg/ml) or vehicle (NaCl, 9 mg/ml) were given *i.p* (200 µl) 7 days before (day -7) and on the same day (day 0) as PAM2 injections. Calvarial bones were harvested eight days after PAM2 injection. Vehicle and PAM2-treated mice were also given anti-RANKL isotype antibodies (rat IgG2a, clone 2A3, Nordic Biosite) using the same protocol as that for anti-RANKL. The isotype antibodies did not affect the loss of bone in PAM2-treated mice as assessed by µCT analysis (data not shown).

### High resolution microCT

High resolution µCT analyses were performed on parietal bones at 14 um resolution using a SkyScan, 1172 (Bruker; 50 kV and 200 uA X-ray source with a 0.5mm Aluminium filter). The scanning angular rotation was 180*°*, and the angular increment was 0.7*°*. Volume of interest was selected from the posterior tip of the frontal bone, extending posteriorly with approximately 8.5 mm in width and 2.67 mm in length, bilateral symmetry along the sagittal suture. Datasets were reconstructed by NRecon (version 1.6.9.8; provided by Bruker) and further analyzed by CTAn (version 1.13.2.1; provided by Bruker).

### Peripheral quantitative computed tomography

Femur bones were dissected and fixed in formalin for 48h and thereafter stored in 70% EtOH until CT scans were performed with the pQCT XCT RESEARCH M (version 4.5B, Norland) and analyzed using the CT software Stratec XCT, Research M pQCT v. 6.20C), as described previously (40). The resolution was 70 µm. The growth plate at distal femur was used as reference line for measuring the metaphysis and diaphysis. The trabecular analysis was positioned in the metaphysis of the femur at a distance proximal from the reference line corresponding to 3% of the total length of the femur. Total bone BMD and trabecular BMD was determined in a single metaphyseal scan, and the inner 45% of the total cross-sectional area was defined as pure trabecular bone region. Cortical bone was determined with the threshold set to 710 mg/cm^3^, and. cortical bone parameters were analyzed in a single scan in the approximate mid-diaphyseal region of the femur, at a distance 36% of the total femur length proximal from the growth plate ^41^. Cortical thickness was calculated by the software as the average thickness using a circular model (41)..

### Static histology

Calvaria and femur were fixed in 4% phosphate-buffered paraformaldehyde, decalcified in 10% EDTA in Tris-buffer pH 6.95, and embedded in paraffin. Sections were stained for TRAP using the Naphtol AS-BI phosphate method together with hematoxylin (Htx) to detect osteoclasts and van Gieson or Masson´s trichrome to visualize collagen fibers. Staining was performed by Histocenter AB, Gothenburg, Sweden according to their accredited protocol.

### Static histomorphometry

Number of osteoclasts and calvarial bone thickness was determined in TRAP/Htx stained sections using the BIOQUANT OSTEO software (version 20.8.60, Bioquant Image Analysis Corporation). TRAP positive osteoclasts on pericranial, endocranial and intracranial (bone marrow) bone surfaces were counted within a distance of 3.5 mm from the mid suture and expressed as number of osteoclasts per mm bone surface. Calvarial bone thickness were measured every 50 μm within a distance of 3.5 mm from the mid suture and expressed as the average calvarial thickness in μm. For static histomorphometry of mineralizing surface in ZA treated mice, mice were injected *i.p.* with calcein (100 μl of 7.5 mg/ml in 2% sodium bicarbonate solution) on day 7 after PAM2 injection. Calvarial bones were dissected eight days after PAM2 injection, fixed in formalin. Metyl methacrylate embedding, and sectioning were performed by PharmaTest Services Ltd. (Turku, Finland). Mineralizing surface per bone surface was determined on pericranial och endocranial surfaces using the Osteomeasure Hisomorphometry System (Osteometrics Inc, Atlanta, USA).

### Dynamic histomorphometry

For dynamic histomorphometry of bone formation parameters in anti-RANKL treated mice, mice were injected *i.p.* with calcein (100 μl of 7.5 mg/ml in 2% sodium bicarbonate solution) on day 2 after PAM2 injection and with alizarin red (100 μl of 15 mg/ml in 2% sodium bicarbonate solution) on day 7 after PAM2 injection. Calvarial bones were dissected eight days after PAM2 injection, fixed in formalin. Metyl methacrylate embedding, sectioning and dynamic histomorphometry were performed by PharmaTest Services Ltd. (Turku, Finland). Dynamic parameters were determined on pericranial and endocranial surfaces using the Osteomeasure Hisomorphometry System (Osteometrics Inc, Atlanta, USA).

### Immunohistochemistry

For detection of proliferating cells, tissue sections of calvaria were stained for the proliferation marker Ki67. After heat-induced antigen retrieval in citrate buffer, pH 6.0, blocking of endogenous peroxidase activity using 3% H_2_O_2_ and blocking of endogenous biotin using an avidin-biotin blocking kit (Vector, Burlingame, CA), sections were incubated with anti-Ki67 antibody (clone SP6, Abcam, Cambridge, UK) diluted 1:100 in PBS at for 60 minutes room temperature. After washings with PBS, sections were incubated with biotinylated goat anti-rat Ig and avidin-biotin-HRP according to the manufacturer´s instructions (Vectastain Elite ABC kit, Vector). 3,3’-diaminobenzidine (DAB) was used as substrate and Htx for nuclear counterstaining.

For detection of Runx2 positive cells, tissue sections of calvaria were immunostained using a rabbit polyclonal anti-Runx2 antibody (ab81357, abcam) and the Mach 3 Rabbit detection kit (RH531L, Biocare Medical), according to the manufacturer´s description. Antigen retrieval was performed in the Rodent decloaker buffer (RD913L, Biocare Medical) in 60°C overnight. Background staining was reduced by incubating sections in 1% H_2_O_2_ for 10 min followed by 10 min in the Sniper solution (BS966L, Biocare Medical). Sections were incubated for 2 hr in room temperature with the antibody diluted 1:50 in the Renaissance Background Reducing Diluent (PD905L, Biocare Medical). Runx2 positive cells were visualized by incubations with the Mach 3 Rabbit probe for 15 min followed by the Mach 3 Polymer for another 15 min and DAB as substrate. Nuclei were counterstained with Htx.

For detection of ALP positive cells, sections of calvaria were immunostained using a rabbit polyclonal anti-Alkaline Phosphatase antibody (ab97384, Abcam) and the EnVision plus HRP Rabbit detection kit (K4003, Dako), according to the manufacturer´s description. Antigen retrieval was performed in citrate buffer (pH=6.0) in 60°C overnight. Background staining was reduced by incubating sections in 3% H_2_O_2_ in protein blocking solution (X0909, Dako) for 20 min. Sections were incubated for 2 hr at room temperature with the antibody diluted 1:30 in antibody diluent (S3022, Dako). ALP-positive cells were visualized with Htx.

For the identification of WNT-activated cells, tissue sections underwent immunostaining using a rabbit recombinant monoclonal anti-ß-catenin antibody (ab32572, Abcam). Antigen retrieval was performed with Dako retrieval solution (S169984-2, Dako), followed by quenching of endogenous peroxidase activity using 3% H_2_O_2_. The primary antibody against ß-catenin was diluted at 1:250 in blocking serum and incubated overnight at 4°C. Subsequently, sections were incubated with a biotinylated secondary antibody (PK-6101, goat anti-rabbit, Vectastain) for 1 hour, and an ABC-AP Kit (AK-5000, Vectastain) for an additional 1 hour, following the manufacturer’s instructions. The liquid red substrate chromogen system (SK-5105, Vectastain) served as the peroxidase substrate for enzymatic amplification. Nuclei were counterstained with Htx.

### Gene expression analysis

**Figure 1:**
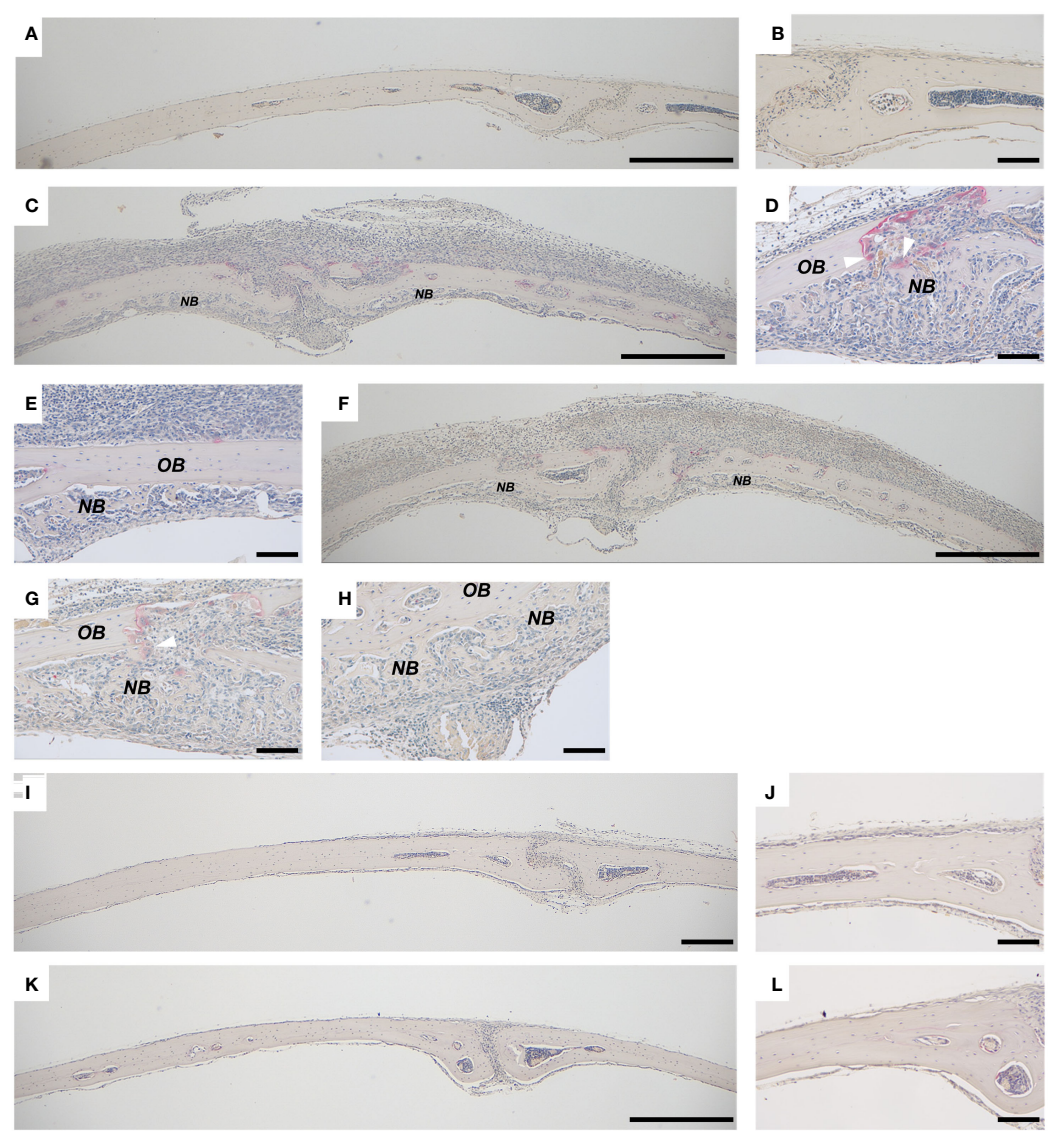
Bone formation in calvarial bones from mice injected with PAM2 or *P. gingivalis* LPS. Calvarial bones from *wild type* C57BL/6J mice injected with vehicle **(A, B)** and new bone formation after injection with PAM2 **(C–E)** or *P. gingivalis* LPS **(F–H)**. No inflammation or new bone formation is observed in calvarial bones from *Tlr2*-deficient mice injected with PAM2 **(I, J)** or *P. gingivalis* LPS **(K, L)**. *NB*, new bone; *OB*, old bone; white arrowheads = mature osteoclasts. Calvarial bones harvested six days after injections. Sections stained for TRAP and counterstained with Htx. Scale bars; **(A, C, F, I, K)**: 500 μm, **(B, D, E, G, H, J, L)** = 100 μm.

Total RNA from skull bones was purified by homogenization of the tissue in Trizol reagent (Life Technologies) followed by purification using the RNeasy^®^ Mini kit (Qiagen). The RNA was reverse transcribed into cDNA using the High Capacity cDNA Reverse Transcription Kit (Applied Biosystems), and real-time PCR analysis was performed using predesigned real-time PCR assays and the StepOnePlus Real-Time PCR system (Applied Biosystems). The following predesigned real-time PCR assays from Applied Biosystems were used for gene expression assays: *Alpl* (Mm00475834_m1), *Col1a1* (Mm00801666_g1), *Bglap* (Mm01741771_g1), *Runx2* (Mm00501580_m1), *Sp7* (*Osterix*; Mm04209856_m1), *Lrp5* (Mm01227476_m1), *Lrp6* (Mm00999795_m1), *Dkk1* (Mm00438422_m1), *Sost* (Mm00470479_m1), *Wnt1* (Mm01300555_g1), *Wnt3a* (Mm00437337_m1), *Wnt4* (Mm01194003_m1), *Wnt5a* Mm00437347_m1), *Wnt7b* (Mm01301717_m1), *Wnt10b* (Mm00442104_m1), *Wnt16* (Mm00446420_m1), *Tnfs11* (*Rankl*; Mm00441908_m1), *Tnfrsf11b* (*Opg*; Mm00435452_m1), *Ctsk* (Mm00484036_m1), *Acp5* (*Trap*; Mm00475698_m1). The amount of RNA purified from the calvarias 5 days after the injection of Pam2 was 3-4 times more than from control injected calvaria or one day after Pam2 injection reflecting the larger numbers of cells in these samples (**Supplementary Figure 1** and histology in **Figure 1**). Three different reference genes (*Rn18s, Gapdh* and *Actb*) were evaluated and normalized to the amount of RNA but were unsuitable as reference genes due to variability in expression per RNA between vehicle and PAM2 injected calvaria, which could be due to the difference in cell composition between vehicle and PAM2 injected calvaria (**Figure 1**). The relative mRNA abundance of each gene was therefore normalized per calvarium. The relative mRNA abundance of each gene was first compared between the different samples (2^-ΔCT^) and then normalized to the total amount of RNA purified from the individual calvaria and expressed as arbitrary units (au).

### *In situ* hybridization (RNAscope)

Fluorescent *in situ* hybridization (FISH) and chromogenic *in situ* hybridization (CISH) were carried out using the RNAscope Multiplex Fluorescent Reagent Kit v2 (ACD; 323100) and RNAscope 2.5 HD Assay – RED kit (ACD; 322360), respectively. Standard RNAscope protocols were implemented following the manufacturer’s instructions with slight adjustments as described previously (42). Target probes (Mm-Wnt7b, ACD, 401131; Mm-SOST-C2, ACD, 410031-C2 an dMm-Runx2-C3, ACD, 414021-C3) were applied and allowed to incubate overnight at 40°C. Imaging was conducted using Nikon spinning disk confocal microscopy and Zeiss Axioscan 7 microscopy. Fluorescent sections were after acquisition of fluorescent images stained with Htx/Eosin to facilitate identification of areas with new bone formation. Chromogenic RNAscope sections were counterstained with Htx.

### Statistics

Numerical data and histograms are expressed as the mean ± S.E.M. An unpaired Student’s *t* test was used to compare two groups. One-way ANOVA followed by Dunnett´s multiple comparison test *vs* CTRL was performed when three groups were included in the experiment. The overall effect of PAM2 (PAM2 *vs* CTRL), anti-RANKL treatment (anti-RL *vs* Vehicle), and their interaction was assessed using 2-way ANOVA, followed by Sidak’s multiple comparison test to evaluate the effect of PAM2 in the absence and presence of anti-RL. The overall effect of PAM2 (PAM2 *vs* CTRL), ZA treatment (ZA *vs* Vehicle), and their interaction was assessed using 2-way ANOVA, followed by Sidak’s multiple comparison test to evaluate the effect of PAM2 in the absence and presence of ZA. The overall effect on calvarial thickness by PAM2, time and their interaction was assessed using 2-way ANOVA followed by Sidak’s multiple comparison test to evaluate the effect of PAM2 at the different time points. Quantitative analyses were made with the investigators blinded to the treatment groups.

## Results

### Inflammation induces new bone formation

**Figure 2:**
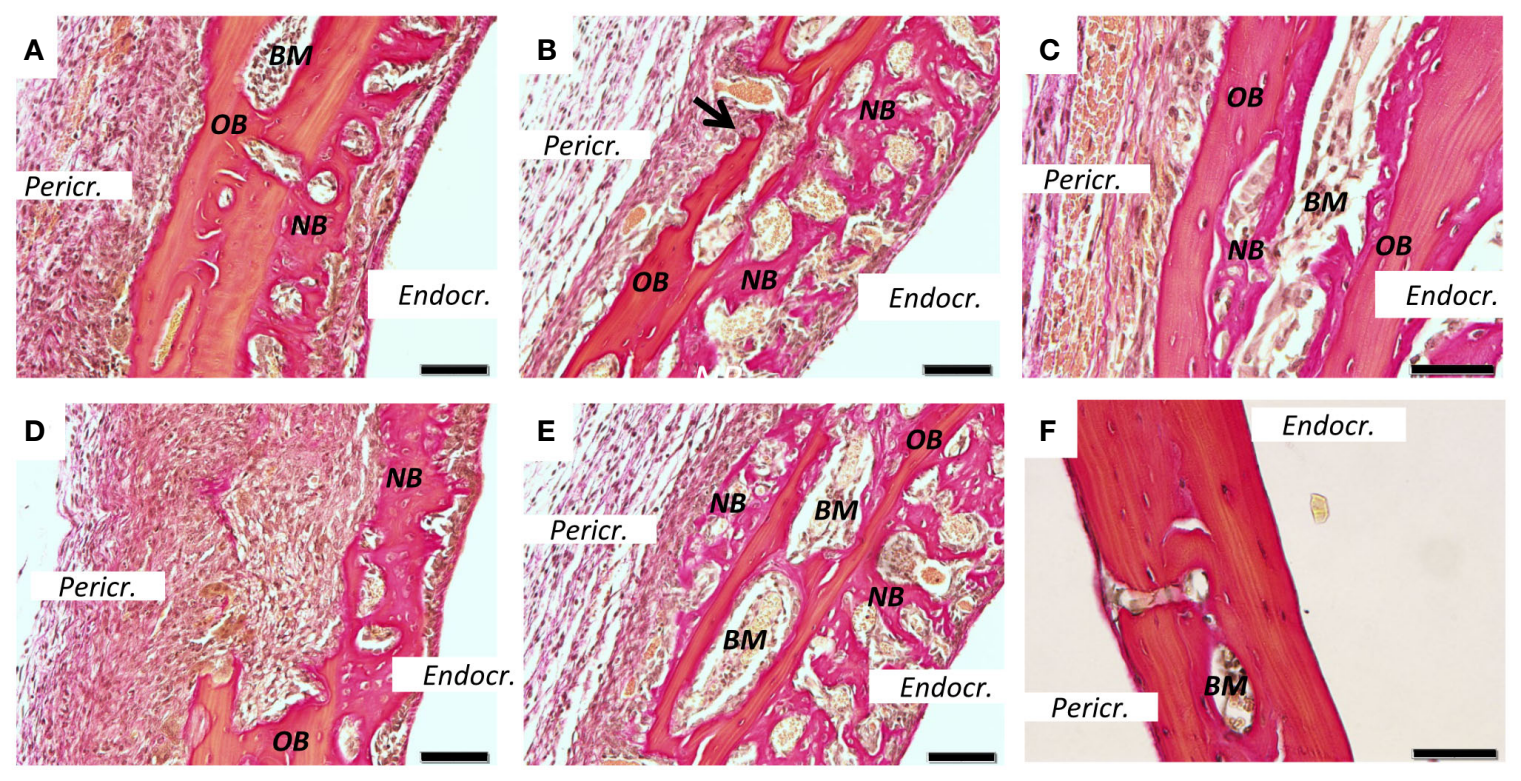
New bone formation induced by PAM2. Calvarial bones five days after injection of PAM2 **(A–E)** or vehicle **(F)**. *OB*, old bone; *NB*, new bone; *Black arrow*, resorption lacunae; *Pericr.*, pericranial side of calvaria. *Endocr.*, endocranial side of calvaria. *BM*, bone marrow. Sections stained with van Gieson. Scale bars; **(A, B, D, E)** = 20 μm, C = 10 μm, F = 50 μm.

**Figure 3:**
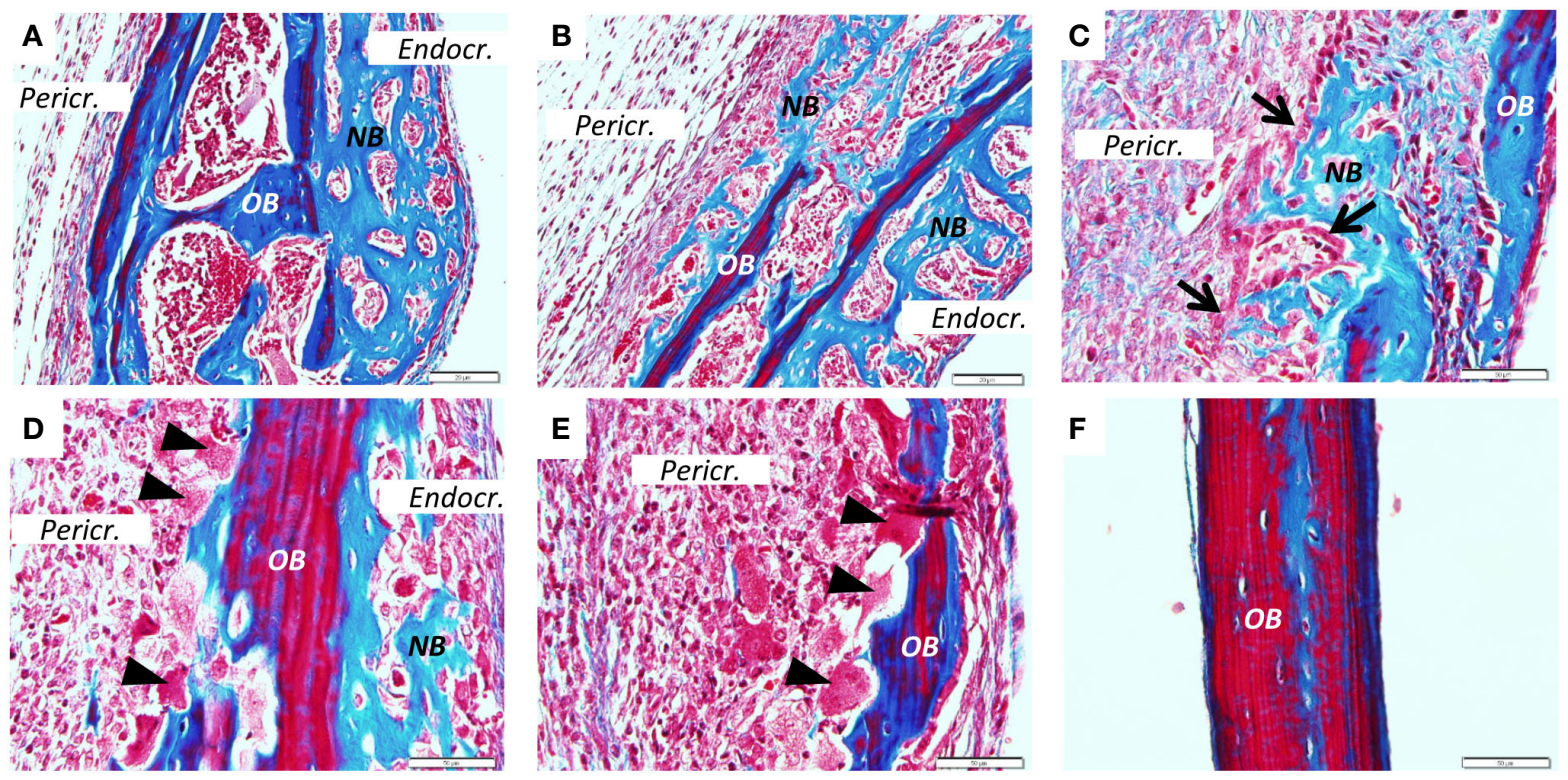
New bone formation induced by PAM2. Calvarial bones five days after injection of PAM2 **(A–E)** compared with vehicle injected bones **(F)**. *OB*, old bone; *NB*, new bone; *Black arrow*, cuboid osteoblast; *Black arrow*heads, osteoclast; *Pericr.*, pericranial side of calvaria; *Endocr.*, endocranial side of calvaria. Sections stained with Masson Trichrome. Scale bars; **(A, B)** = 20 μm, C-F = 50 μm.

We have previously observed that inflammation induced by activation of TLR2 induces excessive local formation of osteoclasts and bone resorption, resulting in extensive loss of bone (38). The periosteal inflammatory reaction is dominated by mononuclear inflammatory cells but contains also some polymorphonuclear leukocytes. We, here, report that the extensive inflammatory process in the periosteum of the mouse calvaria caused by injection of the two TLR2 agonists PAM2 (**Figure 1C**) and *P. gingivalis* LPS (**Figure 1F**) also results in local new bone formation (**Figures 1D, E, G, H**). In contrast, no periosteal inflammation and new bone formation were observed in bones from vehicle treated mice (**Figures 1A, B**). Six days after injection, new bone was formed close to areas where osteoclasts were resorbing old bone (**Figures 1D, G**); in other areas, however, new bone was formed further from osteoclasts (**Figures 1E, H**). No inflammatory reaction or new bone formation was seen after injection with either *P.gingivalis* LPS or PAM2 in mice with deletion of the *Tlr2* gene (**Figures 1I–L**). The newly formed bone appeared as woven-like in a trabecular pattern, distinct from the old lamellar, cortical bone (**Figures 1**–**3**). In bones from mice injected with vehicle, no inflammatory reaction was observed, and all bone tissue was built up by lamellar, cortical bone (**Figures 2F**, **3F**).

We next stained sections from PAM2-stimulated bones with van Gieson (**Figure 2**) or Masson trichrome (**Figure 3**). In the van Gieson stained sections, it was observed that most of the new bone was formed on the endocranial surfaces, in areas previously not resorbed (**Figure 2A**). Some bone was formed close to areas that had been resorbed, although not in the resorption lacunae (**Figure 2B**). Small amounts of new bone formation could be seen in bone marrow compartments (**Figure 2C**). In some areas, new bone bridged over areas which had been extensively resorbed (**Figure 2D**). New bone formation could also be seen on pericranial surfaces, although not to the same extent as on endocranial surfaces; in some areas new bone formation could be seen on both endocranial and pericranial surfaces (**Figure 2E**). In contrast, no inflammation or new bone formation was observed in vehicle treated mice (**Figure 2F**).

Similarly, extensive new bone formation was observed on endocranial surfaces in sections stained with Masson Trichrome (**Figure 3A**). In some areas new bone formation was observed both on endocranial and pericranial surfaces (**Figure 3B**). New bone could also be seen in close proximity to the inflammatory reaction on pericranial surface (**Figure 3C**) with active cuboid osteoblasts on the surface of the newly formed bone (**Figure 3C**). Occasionally, bone resorbing osteoclasts could be seen on the pericranial surface (**Figure 3D**) in areas with new bone formation present on the endocranial surface, whereas in other areas with many osteoclasts and extensive bone resorption no new bone formation was observed (**Figure 3E**). In contrast, no inflammation or new bone formation was observed in vehicle treated mice (**Figure 3F**).

**Figure 4:**
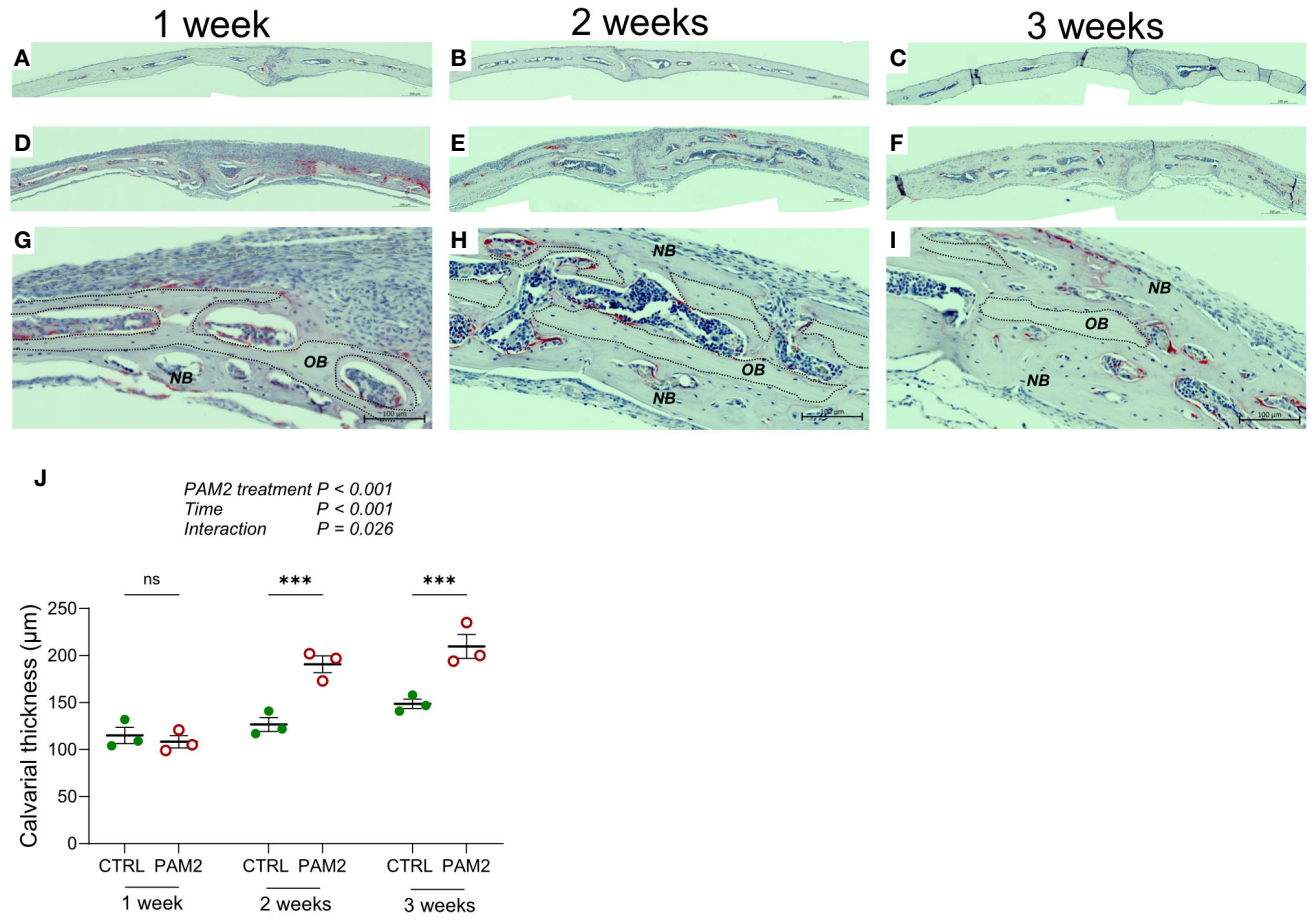
Calvarial thickness increased by PAM2. Calvarial thickness 1-3 weeks following injection of PAM2 **(D–I)** or vehicle (CTRL; **(A–C)**). Photos of TRAP/Htx stained sections **(A–I)** and quantification of calvarial thickness **(J)**. *NB*, new bone; *OB*, old bone; dashed line, border of old bone. Scale bars; **(A–F)**= 200 μm, **(G–I)** = 100 μm. The overall effect on calvarial thickness by PAM2, time and their interaction was assessed using 2-way ANOVA followed by Sidak’s multiple comparison test to evaluate the effect of PAM2 at the different time points. ***P<0.001 *vs* CTRL, ns = non-significant.

The increased new bone formation associated with PAM2 induced inflammation was observed 1, 2 and 3 weeks after initiation of inflammation (**Figures 4A–I**) and resulted in significantly increased thickness of the calvaria, which progressively increased over time (**Figure 4J**). Interestingly, the enhanced thickness at later time points was observed although the inflammatory reaction had attenuated. During the first week after initiation of inflammation, enhanced bone resorption was predominantly observed on pericranial surfaces (**Figure 1**), whereas new bone formation was preferentially seen on endocranial surfaces (**Figures 2**, **3**, **4G**). At later time points, the enhanced thickness of the skull bones was due both to new bone formation on the endocranial surfaces but also to new bone formation in previously extensively resorbed areas on pericranial surfaces (**Figures 4H, I**).

### Local inflammation induces systemic bone loss with no new bone formation

The inflammatory process on the calvaria induced by *P.gingivalis* LPS and PAM2 not only leads to local osteoclast formation and bone loss, but also to systemic bone loss as shown by the robust decrease of total bone mineral density (BMD) and trabecular bone mass in distal femur, with no effect on cortical area or thickness in diaphyseal femur (**Supplementary Figures 2A–D**). The loss of trabecular bone and excessive formation of osteoclasts in distal femur was also evident in histological analysis (**Supplementary Figures 2E–G**). The loss of trabecular bone, however, was not associated with any histological signs of new bone formation.

### Inflammation causes increased bone formation rate

**Figure 5:**
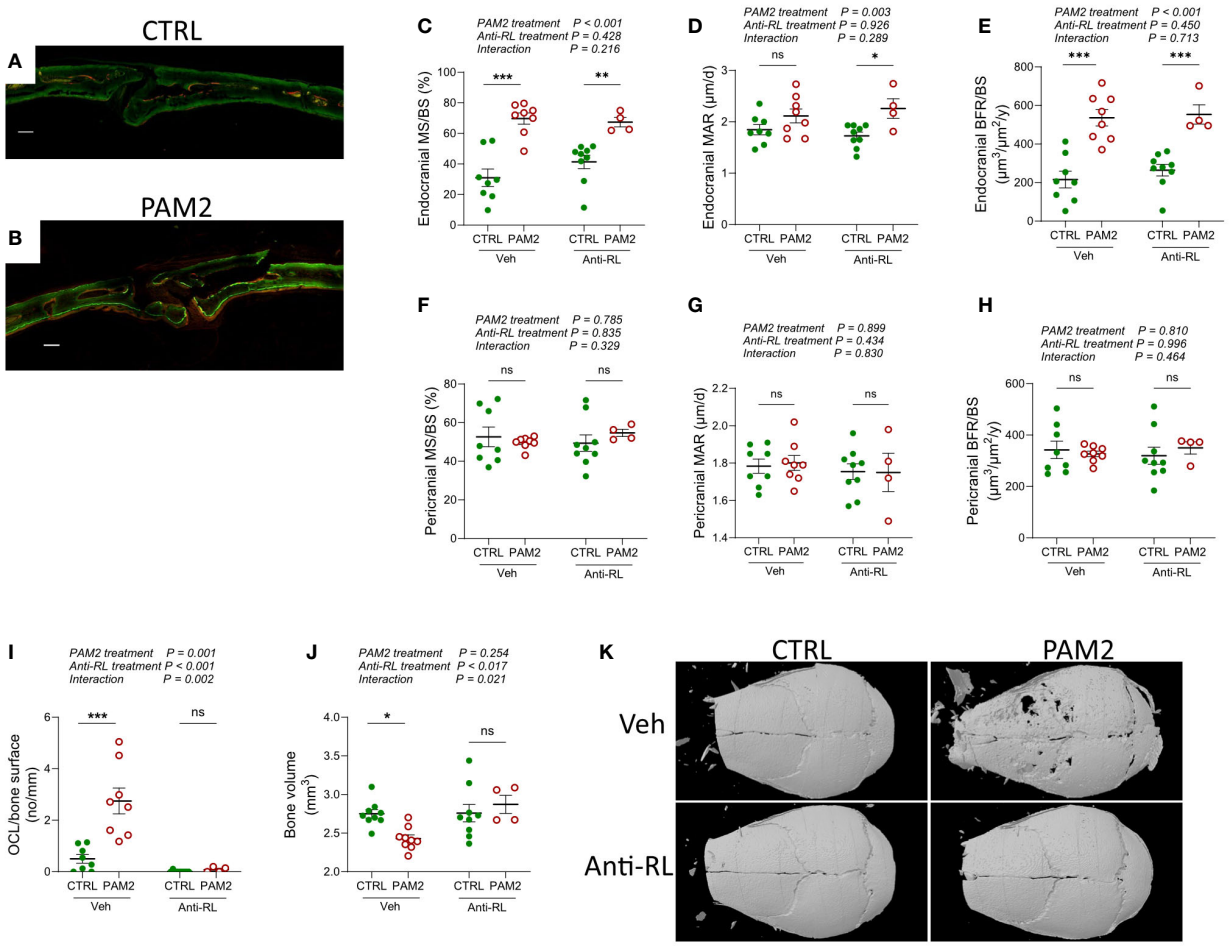
Increased bone formation induced by PAM2 independent on bone resorption. Photos of calcein/alizarin red labeled calvarial bones **(A, B)** and dynamic quantitative histomorphometry **(C–H)** 8 days after injection of PAM2 and/or anti-RANKL (anti-RL). Scale bars (white); **(A, B)** = 100 μm. Number of osteoclast per bone surface 8 days after injection of PAM2 and/or anti-RL **(I)**. Bone volume **(J)** and representative 3-dimensional images **(K)** of calvarial bones analyzed by micro CT 8 days after injection of PAM2 and/or anti-RL. Data presented as individual values with mean ± SEM as vertical lines. The overall effect of PAM2 (PAM2 *vs* CTRL), anti-RANKL treatment (anti-RL *vs* Vehicle), and their interaction was assessed using 2-way ANOVA, followed by Sidak’s multiple comparison test to evaluate the effect of PAM2 in the absence and presence of anti-RL. *P<0.05 *vs* CTRL, **P<0.01 *vs* CTRL, ***P<0.001 *vs* CTRL, ns, non-significant.

To assess bone formation quantitatively, we labelled bone surfaces with fluorescent calcein and alizarin red two and seven days after PAM2 or vehicle injections, and then analyzed labelled surfaces in a fluorescent microscope eight days after PAM2 and vehicle injections (**Figures 5A, B**). This technique allows for quantification of actively mineralizing surfaces as measured by the bone forming osteoblasts (MS/BS) and of rate of mineral apposition reflecting the activity of these osteoblasts (MAR). Using these assessments, the bone formation rate between the two injections can be calculated (BFR/BS). We found that PAM2 induced inflammation caused significantly increased MS/BS and BFR/BS on endocranial surfaces, with no effect on MAR (**Figures 5C–E**), demonstrating that inflammation caused new bone formation by increasing the number of active osteoblasts without affecting their activity. In contrast, no effects on these parameters could be observed on pericranial surfaces (**Figures 5F–H**), which was in line with the morphological observation that pericranial new bone formation was less evident than endocranial new bone formation during the first week after PAM2 injection (**Figures 2**, **3**, **4G**).

### Inflammation-induced new bone formation independent on bone resorption

During physiological remodeling of bone in bone multicellular units, resorption of old bone by osteoclasts is followed by new bone formation in the resorption lacuna due to the release of osteoblast stimulating coupling factors either from the resorbed bone matrix or from the osteoclasts (12). We next investigated if the inflammation induced new bone formation was caused by coupling factors released during bone resorption. Since the cytokine RANKL is crucial for the differentiation of mature, multinucleated, bone resorbing osteoclasts from mononucleated progenitor cells (43) and since the RANKL neutralizing antibody Denosumab inhibits bone erosion in arthritic joints without affecting synovial inflammation in RA patients (44, 45) we used antibodies neutralizing mouse RANKL. The increased MS/BS and BFR/BS caused by PAM2-stimulation on endocranial surfaces was unaffected by treatment with anti-RANKL antibodies (**Figures 5C, E**). Neutralization of RANKL inhibited the increased numbers of osteoclasts (**Figure 5I**) and the robust loss of bone (**Figures 5J, K**) induced by PAM2.

Bisphosphonates, including zoledronic acid, are potent inhibitors of bone resorption by causing cell death of mature osteoclasts through apoptosis (46). Zoledronic treatment abolished the loss of bone induced by PAM2 (**Supplementary Figure 3A**). In mice that received one injection with calcein the day prior to autopsy, labeling was seen on the pericranial side in control mice as expected, which was absent in zoledronic treated controls (**Supplementary Figure 3B**). PAM2-treatment resulted in extensive uptake of calcein which was also observed when PAM2-treated mice were given zoledronic acid. Quantification demonstrated a significantly increased endocranial MS/BS caused by PAM2 which was not affected by co-treatment with zoledronic acid (**Supplementary Figure 3D**).

These observations show that inhibition of bone resorption, caused either by decreasing osteoclast differentiation with anti-RANKL or killing mature osteoclasts with zoledronic acid, does not affect new bone formation induced by inflammation.

### Inflammation induces increased proliferation in areas with new bone formation

**Figure 6:**
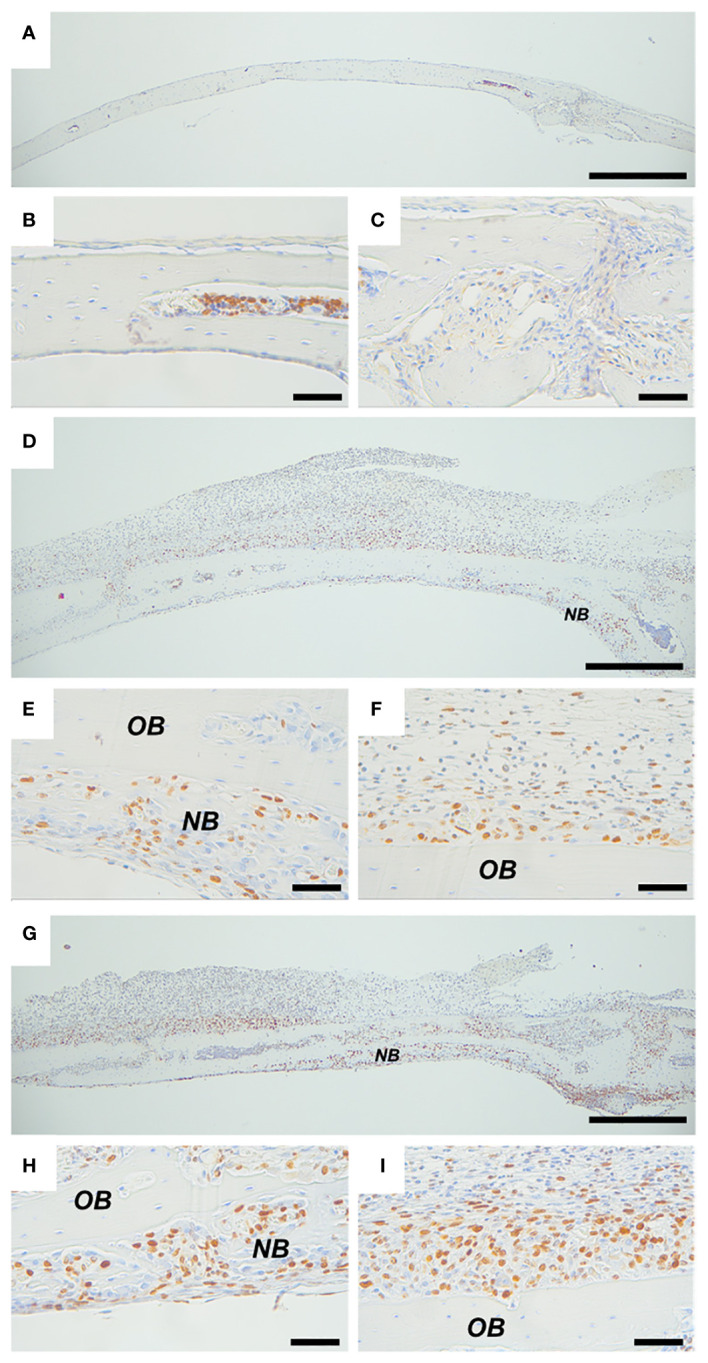
Inflammation induced expression of proliferation marker Ki67. Ki67 staining of calvarial bones from mice injected with vehicle **(A–C)**, *P. gingivalis* LPS **(D–F)** or PAM2 **(G–I)**. *NB*, new bone; *OB*, old bone. Calvarial bones were harvested five days after injection. Scale bars; **(A, D, G)** = 500 μm, **(B, C, E, F, H, I)** = 50 μm.

Ki67 was used as a marker of cell proliferation. In calvarial bones from vehicle-treated mice, several Ki67 positive cells were observed in the bone marrow. In the suture, a few weakly stained cells were present, but no Ki67 positive cells were found on peri- and endocranial surfaces (**Figures 6A–C**). In calvarial bones from *P. gingivalis* LPS- and PAM2-treated mice, Ki67 positive cells were observed on both peri- and endocranial surfaces preferentially in areas close to bone with some few Ki67 positive cells in the pericranial inflammatory infiltrate (**Figures 6D, G**). In areas close to new bone formation, Ki67 positive cells were abundant (**Figures 6E, H**) but several Ki67 positive cells could also be present on pericranial bone surfaces where new bone formation had not yet started (**Figures 6F, I**).

### Inflammation induced expression of genes associated with bone formation

**Figure 7:**
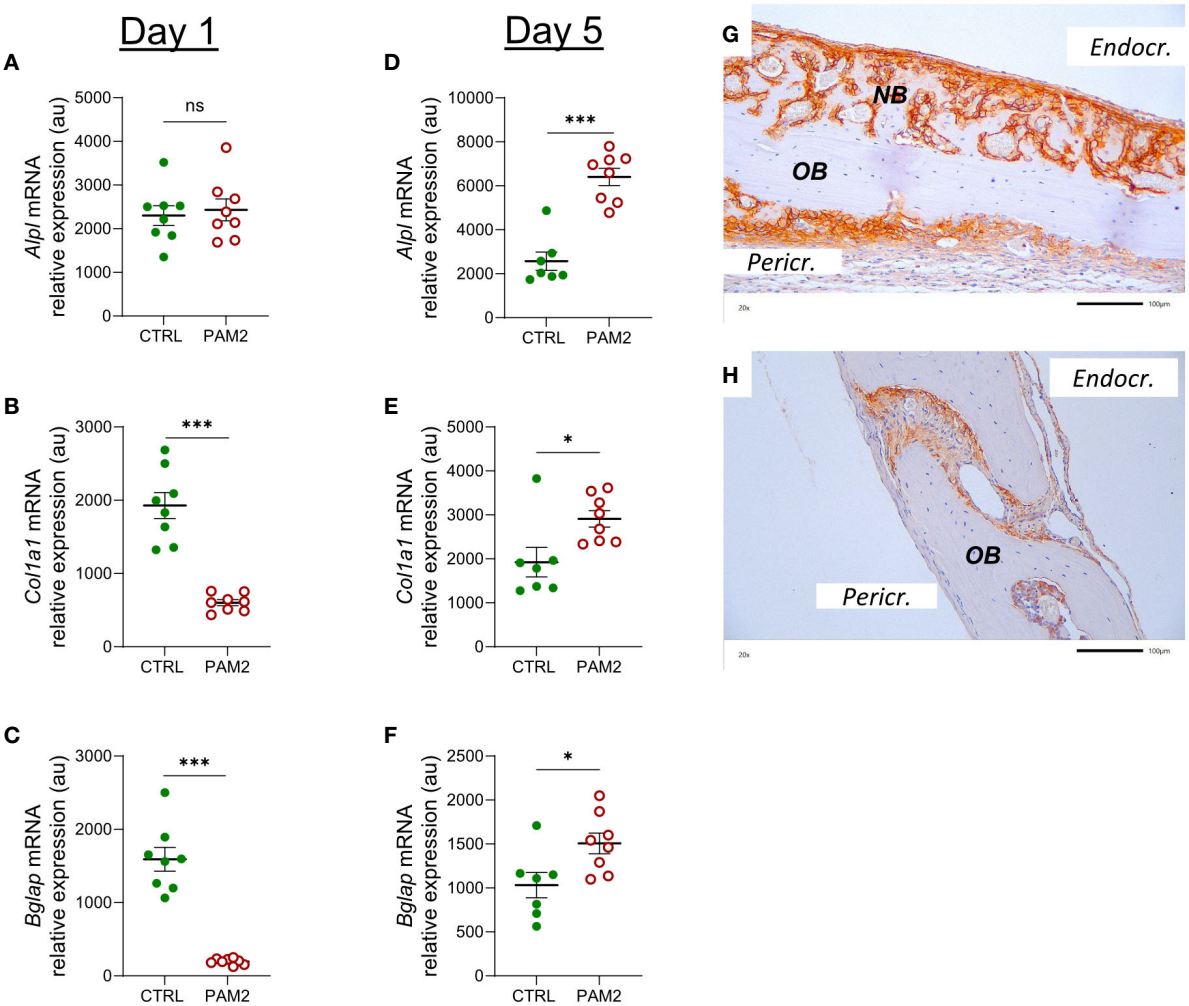
Inflammation induced expression of *Alpl, Col1a1* and *Bglap* associated with bone formation. Gene expression of *Alpl, Col1a1* and *Bglap* analyzed in calvaria dissected one **(A–C)** and five days **(D–F)** after injection of PAM2 or vehicle (CTRL). Immunohistochemical staining for ALP in calvaria dissected 8 days after injection of PAM2 **(G)** or vehicle **(H)**. *OB*, old bone; *NB*, new bone. *Pericr.*, pericranial side of calvaria *Endocr.*, endocranial side of calvaria. Scale bars = 100 μm. Data presented as individual values with mean ± SEM as vertical lines. Statistical analyses were performed using unpaired Student’s *t* test *P<0.05 *vs* CTRL, ***P<0.001 *vs* CTRL, ns, non-significant.

One day after initiation of the inflammatory process *in vivo* by PAM2, the gene expression of *Alpl* was unaffected, whereas the expression of *Col1a1* and *Bglap* (encoding osteocalcin) was decreased (**Figures 7A–C**). Four days later, the expression of *Alpl*, *Col1a1*and *Bglap* was increased by the PAM2 injection (**Figures 7D–F**), in line with the histological observations demonstrating new bone formation at this time point.

In agreement with the mRNA expression data, increased protein expression of ALP was observed in areas with new bone formation, mainly on endocranial surfaces (**Figure 7G**), but also in some areas on pericranial surfaces (**Figure 7G**). In bones from vehicle treated mice, some ALP positive cells could be seen in sutures (**Figure 7H**).

**Figure 8:**
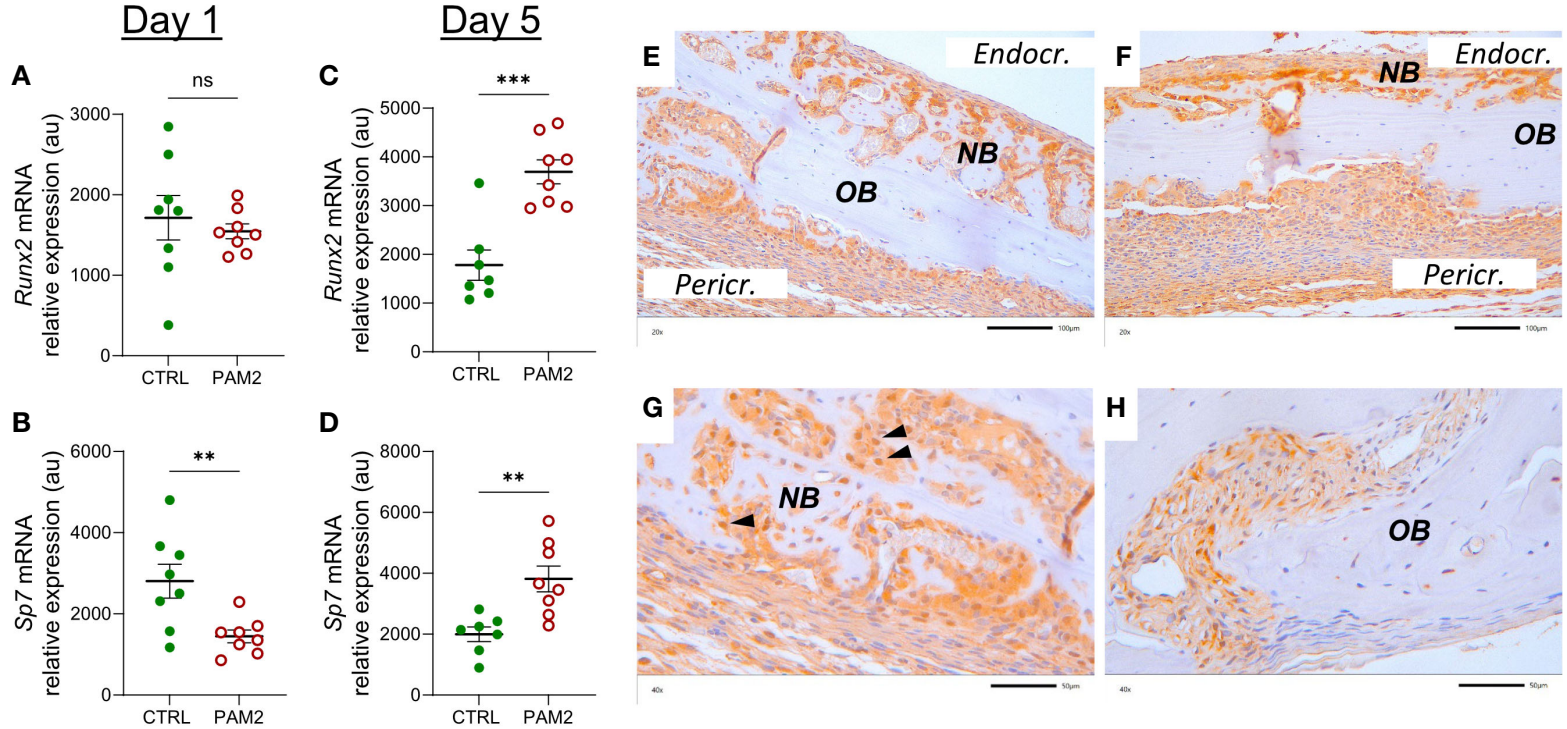
Inflammation induced expression of *Runx2* and *Sp7*. Gene expression of *Runx2* and *Sp7* analyzed in calvaria dissected one **(A, B)** and five days **(C, D)** after injection of PAM2 or vehicle (CTRL). Immunohistochemical staining for Runx2 protein in calvaria dissected eight days after injection of PAM2 **(E–G)** or vehicle **(H)**. *OB*, old bone; *NB*, new bone, black arrowheads, nuclear staining for Runx2 *Pericr.*, pericranial side of calvaria *Endocr.*, endocranial side of calvaria. Scale bars; **(E, F)** = 100 μm, **(G, H)** = 50 μm. Data presented as individual values with mean ± SEM as vertical lines. Statistical analyses were performed using unpaired Student’s *t* test. **P<0.01 *vs* CTRL, ***P<0.001 *vs* CTRL, ns, non-significant.

The mRNA expression of *Sp7* (encoding osterix) was decreased one day after initiation of the inflammation whereas that of *Runx2* was unaffected (**Figures 8A, B**). On day five after initiation of inflammation, the mRNA of both *Runx2* and *Sp7* was upregulated (**Figures 8C, D**). In agreement with the mRNA expression data, increased protein expression of *Runx2* was observed in osteoblasts from areas with new bone formation (**Figures 8E–G**); in some cells, the nuclei were strongly stained for Runx2 (**Figure 8G**). In bones from vehicle treated mice, some Runx2 positive cells were seen in sutures (**Figure 8H**).

In agreement with our previous observations (38), PAM2-induced inflammation also increased the expression of genes associated with bone resorption. The mRNA expression of *Tnfsf11* (encoding RANKL) was upregulated by PAM2 both at day 1 and 5 (**Supplementary Figures 4A, D**). Also the expression of *Tnfrsf11b* (encoding OPG) was increased (**Supplementary Figures 4B, E**) but the ratio *Tnfsf11*/*Tnfrsf11b* was significantly enhanced in the PAM2 treated bones compared with vehicle treated bones at both time points (**Supplementary Figures 5C, F**), resulting in increased expression of *Ctsk* (encoding cathepsin K) and *Acp5* (encoding TRAP) in the PAM2 treated bones (**Supplementary Figures 4G–J**).

### Increased expression of WNT signaling components

**Figure 9:**
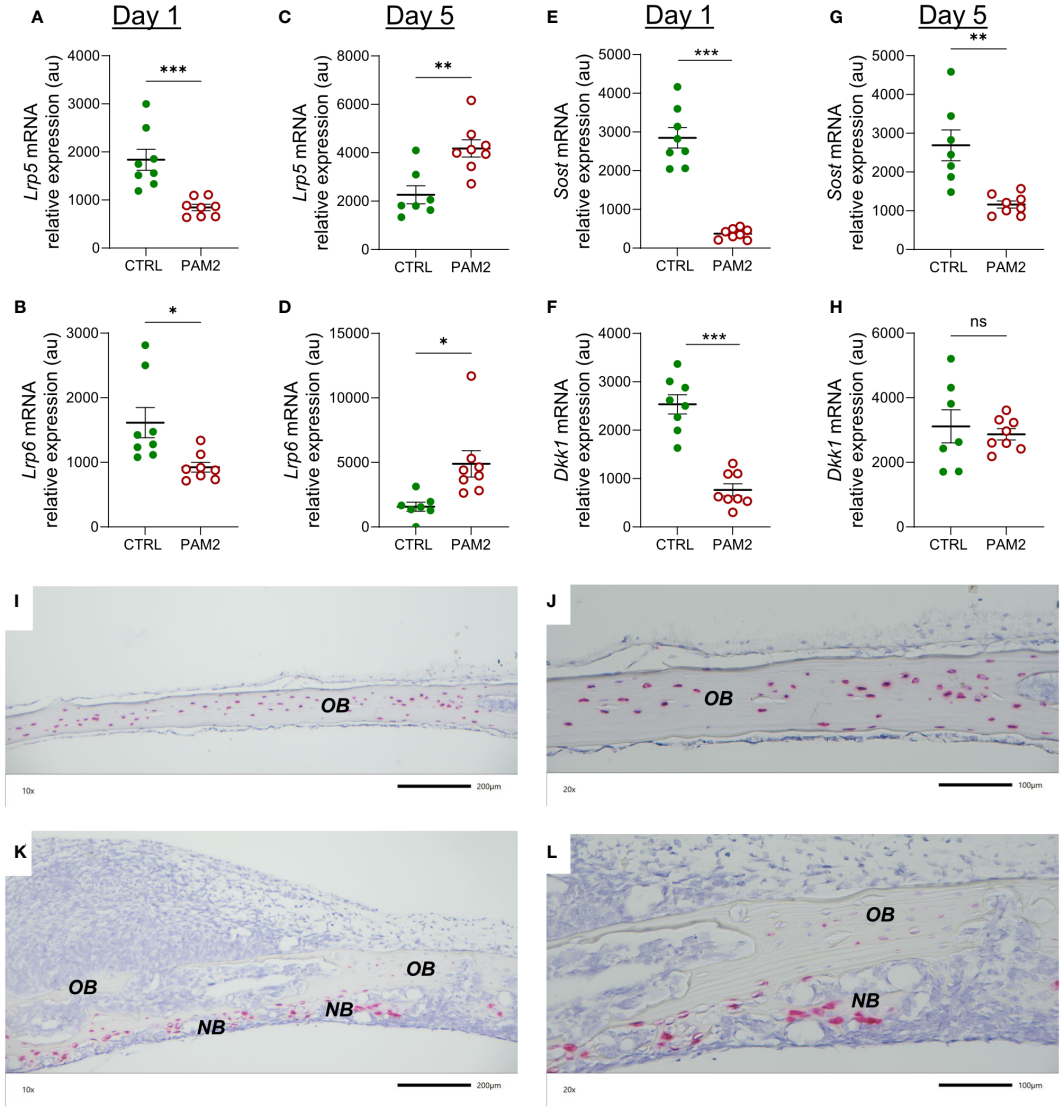
The effect of PAM2 on WNT signaling components. Gene expression of *Lrp5, Lrp6, Sost* and *Dkk1* analyzed in calvaria dissected one **(A, B, E, F)** and five days **(C, D, G, H)** after injection of PAM2 or vehicle (CTRL). *In situ* hybridization for expression of *Sost* five days after injection of vehicle (CTRL) **(I, J)** or PAM2 **(K, L)**. *OB*, old bone; *NB*, new bone. Scale bars; **(I, K)** = 200 μm, **(J, L)** = 100 μm. Data presented as individual values with mean ± SEM as vertical lines. Statistical analyses were performed using unpaired Student’s *t* test. *P<0.05 *vs* CTRL, **P<0.01 *vs* CTRL, ***P<0.001 *vs* CTRL, ns, non-significant.

Canonical and non-canonical WNT signaling has been shown to be crucial for bone formation (47, 48). We assessed several components in this system for their expression in the PAM2-stimulated calvarial bones. Lipoprotein receptor-related proteins 5 and 6 (LRP5, LRP6) are co-receptors for WNT ligands in canonical signaling. The mRNA expression of *Lrp5* and *Lrp6* was decreased at day 1 and increased at day 5 in the PAM2 treated bones (**Figures 9A–D**).

Dkk1 and sclerostin inhibit canonical WNT signaling by binding to LRPs. The mRNA expression of *Dkk1* and *Sost* (encoding sclerostin) was decreased at day 1 by PAM2 treatment; at day 5, the mRNA expression of *Sost*, but not *Dkk*,*1* was still inhibited (**Figures 9E–H**).

Using *in situ* hybridization, it was observed that the majority of osteocytes in bones from vehicle treated mice expressed *Sost* mRNA while very few cells on pericranial or endocranial surfaces expressed *Sost* (**Figures 9I, J**). In bones from mice with pericranial inflammation harvested five days after PAM2 injection, the osteocytic expression of *Sost* in old bone was substantially decreased (**Figures 9K, L**). Interestingly, however, several of the osteoblasts in areas with new bone formation expressed *Sost* mRNA (**Figures 9K, L**).

**Figure 10:**
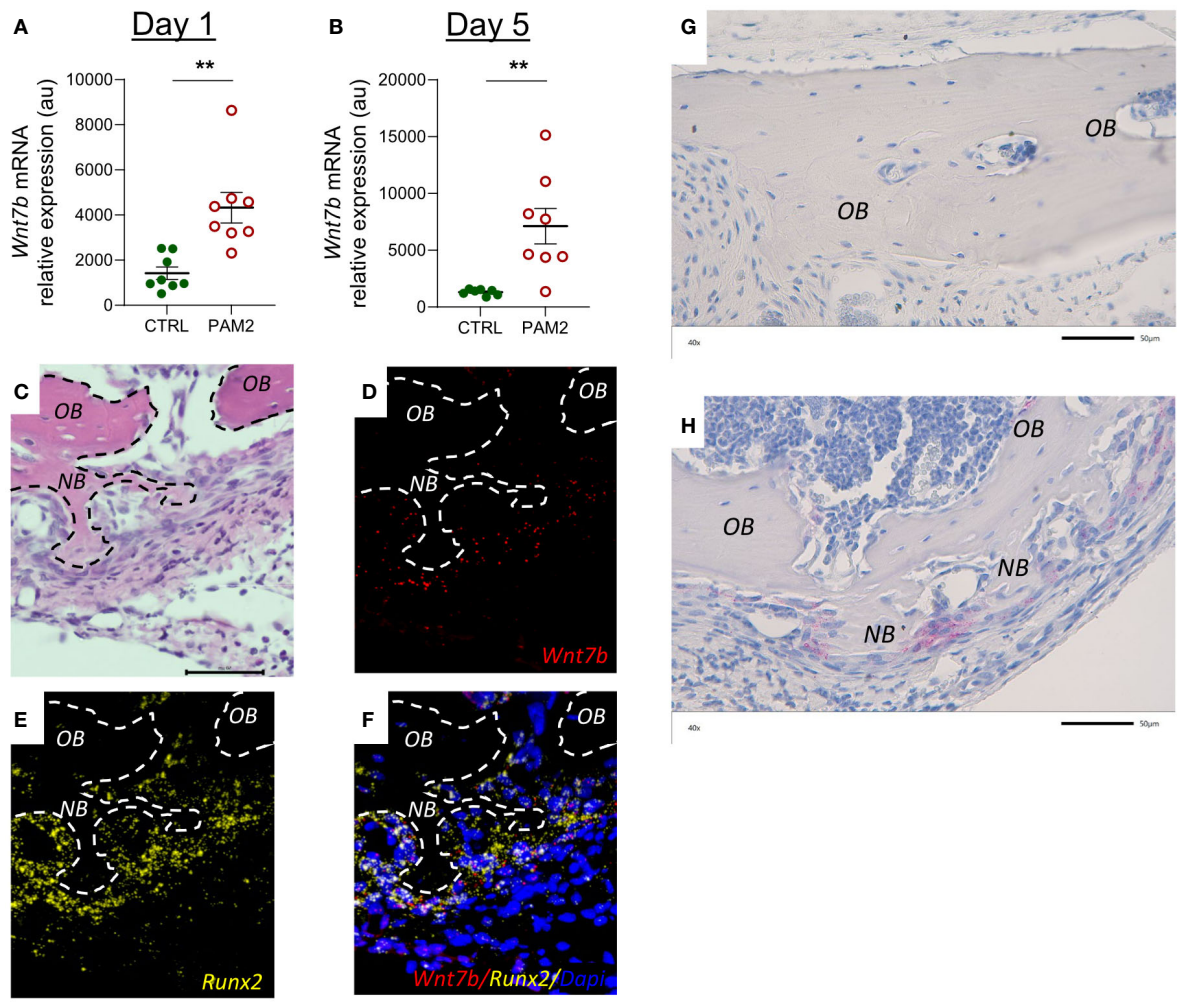
Inflammation induced expression of Wnt7b and β-Catenin. Gene expression of *Wnt7b* analyzed in calvarias dissected one **(A)** and five days **(B)** after injection of PAM2 or vehicle (CTRL). Htx/Eosin staining **(C)** and *in situ* hybridization for expression of *Wnt7b* [red, **(D)**], *Runx2* [yellow, **(E)**] and overlay of *Wnt7b, Runx2* and Dapi staining **(F)** of calvaria dissected five days after injection of PAM2. Immunohistochemical staining for β-Catenin (pink) in calvaria dissected five days after injection of vehicle (CTRL) **(G)** or PAM2 **(H)**. *OB*, old bone; *NB*, new bone. Scale bars = 50 μm. Data presented as individual values with mean ± SEM as vertical lines. Statistical analyses were performed using unpaired Student’s *t* test, **P<0.01 *vs* CTRL.

WNT constitutes a large family of 19 soluble ligands which can regulate the activities of a variety different cell types, including bone cells. It is not known, yet, which WNTs are most important in bone physiology and pathology, but some WNTs have been shown to stimulate osteoblast differentiation and bone formation (47, 48). In PAM2 treated bones, we observed that the mRNA expression of *Wnt7b* at day 1 and 5 was significantly increased (**Figures 10A, B**). The mRNA expression of *Wnt1*, *Wnt10b* and *Wnt16* was decreased at day 1, whereas *Wnt4* was increased and *Wnt5*a was unaffected (**Supplementary Figures 5A–E**). At day 5, the mRNA expression of *Wnt16* was upregulated by PAM2, whereas *Wnt1*, *Wnt5a*, and *Wnt10b* was not changed and *Wnt4* decreased (**Supplementary Figures 5F–J**). *Wnt3*a mRNA was undetectable at both time points.

*In situ* hybridization demonstrated that *Wnt7b* mRNA was highly expressed in areas with new bone formation in *Runx2* mRNA expressing osteoblasts (**Figures 10C–F**). Immunohistochemistry analysis demonstrated that cells close to areas with new bone formation expressed ß-catenin (**Figures 10G, H**).

## Discussion

It is commonly regarded that inflammatory processes cause bone loss by increasing bone resorption and decreasing bone formation (7, 8, 10, 11). We, here, show that inflammatory processes on the top of calvaria can cause both increased bone resorption and increased new bone formation. These observations were made after stimulation of the innate immune system by activating TLR2, by local *s.c*. injection of either *P. gingivalis* LPS or the synthetic ligand PAM2. Formation of new bone was seen in some areas close to the presence of osteoclasts and excessive bone resorption, but in other areas the new bone was clearly distant from osteoclasts, indicating that the skeletal reaction was due to increased bone modeling rather than to enhanced remodeling (12, 49). A major part of new bone was observed at endocranial surfaces whereas bone resorption was predominantly observed on pericranial surfaces close to the inflammatory process. Some areas with new bone formation without any sign of resorption were observed in bone marrow compartments. These observations support the view that new bone was not formed as part of remodeling. Although new bone was not formed in resorption lacunae, such as in physiological remodeling, the possibility exists that new bone formation was caused by coupling factors released either from the bone matrix or from the osteoclasts and acting in areas remote from the resorption lacunae. However, we found that new bone formation was observed also when osteoclast formation and bone resorption was blocked by anti-RANKL or zoledronic acid.

The formation of new bone seems to be mainly a local effect in the skull bones since, although the skull bone inflammation results also in systemic loss of trabecular bone, no signs of new bone formation were observed in the long bones.

The type of bone formed was mainly an osteocyte-rich woven bone where active, cuboidal osteoblasts were observed at the bone surfaces. In most areas, new bone was formed in an irregular pattern which was not caused by the stimulation of existing, mature osteoblasts but rather the stimulation of proliferation and differentiation of cells in the osteoblast-lineage. Support for the view that new bone formation was associated with increased cell proliferation was the observation that many Ki67 positive cells were present in areas where new bone was formed.

One day after initiation of inflammation, the mRNA expression of bone matrix proteins such as *Col1a1* and *Bglap*, and the osteoblastic transcript factor *Sp7* was decreased, in line with observations by others showing that inflammation can inhibit bone formation (13, 15). The expression of these genes, as well as the mRNA expression of the osteoblastic transcription factor *Runx2* and *Alpl*, a cellular marker of active osteoblasts, were significantly upregulated five days later when a robust pericranial inflammatory process was observed. In agreement with these findings, the majority of cells in the area with new bone formation were positively stained for ALP and Runx2 proteins. Some osteoblast nuclei were strongly stained for Runx2, indicating nuclear translocation of this osteoblastic transcription factor, which demonstrates that these osteoblasts were transcriptionally activated. These data suggest that although pericranial inflammation seemed to decrease bone formation initially, this inhibition was transient and that the inflammatory process has the capability to induce stimulation of new bone formation.

Supporting our morphological evidence of new bone formation, we also analyzed bones labelled with the fluorescent dyes calcein and alizarin red. The dynamic histomorphometic analysis demonstrated that the pericranial inflammatory process robustly enhanced bone formation on endocranial surfaces during the first week after initiation of inflammation, as assessed by increased BFR/BS. This was due to increased numbers of osteoblasts (MS/BS) with no effect on their bone forming activity (MAR). Using this technique, no effect on bone formation on pericranial surfaces was noted which reflects that new bone formation on these surfaces during the first week was much less than on endocranial surfaces using histological assessments.

Formation of new bone was sustained after the first week as shown by the finding that the width of the skull bones from mice with pericranial inflammation increased progressively during the following two weeks compared to vehicle treated mice although the inflammatory reaction had attenuated. This finding show that the enhanced osteoblast activity did not require the continuous presence of inflammation once the cells had been activated. Interestingly, the enhanced width was not due entirely to endocranial bone formation since at the later time points new bone had formed above the extensive resorption lacunae observed on pericranial surfaces.

Increased new bone formation, associated with inflammation induced osteoclastogenesis and bone loss, has also been observed in murine arthritis. Mice overexpressing hTNF exhibit polyarthritis, cartilage breakdown and local bone resorption due to synovitis (16, 35). Interestingly, these studies observed increased osteoblast activity and new bone formation at endosteal surfaces of cortical bone but only in close vicinity of where bone marrow inflammation could be seen (35). The degree of new bone formation in the hTNF model was, however, much less than the abundant formation of woven bone observed in our model.

In patients with RA, areas with active osteoblasts depositing wide osteoid seams on endosteal surfaces of cortical bone have been observed (24). On these endosteal surfaces, no osteoclasts were seen. Signs of bone formation were also found in these patients on trabecular bone, which exhibited not only increased number of osteoclasts but also enhanced number of osteoblasts, as compared with healthy normal individuals.

As discussed above, new bone formation in resorption lacunae during physiological remodeling is stimulated by coupling factors released by osteoclasts (12). In the present experiments, the formation of new bone was not located in the resorption lacunae. However, since activation of periosteal inflammation in the skull bones results in extensive formation of bone resorbing osteoclasts (38) it might be that release of coupling factors may have reached osteoblast progenitors remote from the resorption lacunae. To test this hypothesis, we blocked osteoclast formation and bone resorption by treating the mice with either anti-RANKL antibodies or by injecting zoledronic acid and found that also under these circumstances new bone formation was induced by the periosteal inflammation. This finding suggests that the formation of new bone was induced by the inflammatory process. Signaling molecules from the pericranial inflammatory reaction might act locally to induce pericranial new bone formation but also reach the endocranial surfaces via sutures and microscopic vascular channels crossing the skull bone to induce endocranial new bone formation (50, 51).

WNT signaling plays an important role for bone formation and bone mass as demonstrated by observations in humans and in mice with either gain- or loss-of-function mutations or deletions of genes involved in LRP/WNT/Frizzled signaling (47, 48). Canonical WNT signaling is stimulated by binding of WNT ligands to the co-receptors LRP5 or LRP6 followed by formation of a heterotrimeric complex due to association with transmembrane receptors Frizzled and downstream signaling. Binding sites for DKK1 and sclerostin are present in the extracellular domains of LRPs. When occupied by DKK1 or sclerostin the binding of the co-receptors to Frizzled is inhibited and WNT canonical signaling blocked (47, 48). Sclerostin is preferentially expressed by osteocytes, whereas DKK1 is expressed by several cell types. The crucial importance of sclerostin for bone formation is demonstrated by the excessive formation of bone in patients with von Buchems disease and sclerosteosis harboring loss-of-function mutations in the *SOST* gene (52, 53) and by the use of anti-sclerostin antibodies as an anabolic treatment for patients with osteoporosis (54, 55). In the current study, five days after initiation of inflammation, mRNA expression of *Lrp5* and *Lrp6* was significantly increased compared with the expression in vehicle treated bone as assessed by qPCR. Furthermore, we observed robust decreased mRNA expression of the genes encoding the inhibitors DKK1 and sclerostin using qPCR analyses. In line with the qPCR analysis, *in situ* hybridization demonstrated substantially decreased expression of *Sost* in the osteocytes present in remaining old bones from mice with periosteal inflammation. Interestingly, several of the osteoblasts in areas with new bone formation expressed *Sost* indicating that these cells had started to be transformed to osteocytes. Most likely, *Sost* is upregulated as a negative feedback response to the abundant new bone formation. These observations suggest that inflammatory induced recruitment and differentiation of osteoblasts in our model was caused by canonical WNT signaling.

There are 19 different WNT ligands (47, 48). Several WNTs have been shown to be expressed by bone cells and to affect bone mass through effects on osteoblasts and osteoclasts. The crucial role of WNTs on bone mass was demonstrated by the findings that inhibition of the intracellular processing needed for WNT secretion by porcupine inhibition or deletion of the gene encoding Wntless results in decreased bone formation and bone mass (56, 57). However, much remains to be known about the specific physiological and pathophysiological roles of the different WNTs in bones. We have previously reported that WNT16 is produced by cortical osteoblasts and enhances bone mass mainly by inhibiting osteoclast differentiation through a paracrine effect on osteoclast progenitors but also due to a paracrine/autocrine stimulatory effect on osteoblasts (37). Based upon large scale genome-wide association studies it has been found that WNT16 is preferentially important for forearm fractures with no association to hip fractures (42), indicating that different WNTs have site-specific effect on bone mass and fracture susceptibility. We analyzed the expression of WNTs known to stimulate osteoblasts in bulk RNA from skull bones and found that the mRNA expression of *Wnt7b* was robustly enhanced both one and five days after initiation of inflammation, whereas *Wnt1*, *Wnt4*, *Wnt5a*, *Wnt10* and *Wnt16* mRNA expression was not consistently regulated and *Wnt3a* was not detectable. To assess which cells expressed *Wnt7b* we performed fluorescent *in situ* hybridization and observed that *Wnt7b* was expressed by *Runx2*-expressing cells in areas of new bone formation. There are several lines of evidence suggesting that Wnt7b is important for new bone formation. Wnt7b stimulates osteoblastic differentiation of the embryonic mesenchymal cell line C3H10T1/2 and the bone marrow stromal cell line ST-3 (58, 59). Overexpression of *Wnt7b* in osteoblasts increases the number and activity of osteoblastic cells causing high bone mass (60, 61). Wnt7b enhances self-renewal and osteogenic commitment of bone marrow mesenchymal stem cells (62). Increased levels of Wnt7b have been observed in experimentally induced fracture healing (62) and induced expression of *Wnt7b* enhances bone formation in a mouse calvarial defect model (61). These findings indicate that Wnt7b is likely to have an important anabolic role in our model of inflammation-induced bone formation. Moreover, Wnt7b may also enhance bone mass by inhibiting osteoclast differentiation (63). The effect by Wnt7b in several of these studies has been attributed to non-canonical WNT signaling. There are, however, examples where canonical WNT signaling has been associated with stimulatory effects by Wnt7b in osteoblasts (64) as well as in other cell types (65). We found that *Lrp5* and *Lrp6* mRNA were upregulated and that *Sost* and *Dkk1* were downregulated by inflammation in the calvaria which indicates that canonical WNT signaling was involved. An important hallmark of WNT canonical signaling is the stabilization of ß-catenin (47, 48). Further support for the involvement of canonical WNT signaling is that positive staining for ß-catenin was demonstrated in osteoblasts in areas with new bone formation but final proof for which signaling pathway is important must await more detailed analysis.

In summary, we here show that inflammation can not only stimulate local and systemic osteoclast formation and bone loss but also concomitantly local bone formation. This response was independent on resorption suggesting that the formation of new bone was induced by the inflammatory process per se and should be regarded as inflammatory induced modeling rather than remodeling. The effect was associated with increased expression of the osteoblastic transcription factors Runx2 and osterix and with effects on molecules involved in canonical WNT signaling such as downregulation of the inhibitors Dkk1 and Sost, upregulation of the osteoanabolic ligand Wnt7b and stabilization of ß-catenin. The stimulation of bone formation is most likely part of the resolution of inflammation, but likely also reflects the process by which inflammation induces healing of skeletal fractures. Knowledge of the cellular and molecular mechanisms causing bone formation in inflammatory process may reveal new targets for stimulating bone formation in diseases with local and systemic bone loss such as osteoporosis, osteogenesis imperfecta, periodontal disease and arthritis.

## Data availability statement

The raw data supporting the conclusions of this article will be made available by the authors, without undue reservation.

## Ethics statement

The animal study was approved by Ethical Committees for Animal Research at University of Gothenburg and University of Umeå. The study was conducted in accordance with the local legislation and institutional requirements.

## Author contributions

PH: Writing – review & editing, Visualization, Investigation, Formal analysis, Methodology, Data curation, Conceptualization. AK: Writing – review & editing, Investigation. AW: Writing – review & editing, Visualization, Methodology, Investigation. PL: Writing – review & editing, Investigation. CE: Writing – review & editing, Resources, Investigation. VL: Writing – review & editing, Investigation. PW: Writing – review & editing, Investigation. JW: Writing – review & editing, Investigation. LL: Writing – review & editing, Visualization, Investigation. CL: Writing – review & editing, Investigation. PS: Writing – review & editing, Conceptualization. SM-S: Methodology, Funding acquisition, Conceptualization, Writing – review & editing. UL: Writing – original draft, Supervision, Resources, Project administration, Methodology, Data curation, Conceptualization.

## References

[1] Harre U Schett G. Cellular and molecular pathways of structural damage in rheumatoid arthritis. Semin Immunopathol. (2017) 39:355–63. doi: 10.1007/s00281-017-0634-0 28597065

[2] Andreev D Kachler K Schett G Bozec A. Rheumatoid arthritis and osteoimmunology: The adverse impact of a deregulated immune system on bone metabolism. Bone. (2022) 162:116468. doi: 10.1016/j.bone.2022.116468 35688359

[3] Cekici A Kantarci A Hasturk H Van Dyke TE. Inflammatory and immune pathways in the pathogenesis of periodontal disease. Periodontology 2000. (2014) 64:57–80. doi: 10.1111/prd.12002 24320956 PMC4500791

[4] Zhou M Graves DT. Impact of the host response and osteoblast lineage cells on periodontal disease. Front Immunol. (2022) 13:998244. doi: 10.3389/fimmu.2022.998244 36304447 PMC9592920

[5] Cobelli N Scharf B Crisi GM Hardin J Santambrogio L. Mediators of the inflammatory response to joint replacement devices. Nat Rev Rheumatol. (2011) 7:600–8. doi: 10.1038/nrrheum.2011.128 21894210

[6] Fransson C Tomasi C Pikner SS Grondahl K Wennstrom JL Leyland AH. Severity and pattern of peri-implantitis-associated bone loss. J Clin Periodontol. (2010) 37:442–8. doi: 10.1111/j.1600-051X.2010.01537.x 20507368

[7] Walsh NC Gravallese EM. Bone remodeling in rheumatic disease: a question of balance. Immunol Rev. (2010) 233:301–12. doi: 10.1111/j.0105-2896.2009.00857.x 20193007

[8] Schett G Gravallese E. Bone erosion in rheumatoid arthritis: mechanisms, diagnosis and treatment. Nat Rev Rheumatol. (2012) 8:656–64. doi: 10.1038/nrrheum.2012.153 PMC4096779 23007741

[9] Bartold PM Cantley MD Haynes DR. Mechanisms and control of pathologic bone loss in periodontitis. Periodontology. (2000) 2010:53:55–69. doi: 10.1111/prd.2010.53.issue-1 20403105

[10] Souza PP Lerner UH. The role of cytokines in inflammatory bone loss. Immunol Invest. (2013) 42:555–622. doi: 10.3109/08820139.2013.822766 24004059

[11] Mbalaviele G Novack DV Schett G Teitelbaum SL. Inflammatory osteolysis: a conspiracy against bone. J Clin Invest. (2017) 127:2030–9. doi: 10.1172/JCI93356 PMC5451216 28569732

[12] Sims NA Martin TJ. Osteoclasts provide coupling signals to osteoblast lineage cells through multiple mechanisms. Annu Rev Physiol. (2020) 82:507–29. doi: 10.1146/annurev-physiol-021119-034425 31553686

[13] Walsh NC Reinwald S Manning CA Condon KW Iwata K Burr DB. Osteoblast function is compromised at sites of focal bone erosion in inflammatory arthritis. J Bone Miner Res. (2009) 24:1572–85. doi: 10.1359/jbmr.090320 19338457

[14] Kuratani T Nagata K Kukita T Hotokebuchi T Nakasima A Iijima T. Induction of abundant osteoclast-like multinucleated giant cells in adjuvant arthritic rats with accompanying disordered high bone turnover. Histol Histopathol. (1998) 13:751–9. doi: 10.14670/HH-13.751 9690133

[15] Diarra D Stolina M Polzer K Zwerina J Ominsky MS Dwyer D. Dickkopf-1 is a master regulator of joint remodeling. Nat Med. (2007) 13:156–63. doi: 10.1038/nm1538 17237793

[16] Zwerina J Tuerk B Redlich K Smolen JS Schett G. Imbalance of local bone metabolism in inflammatory arthritis and its reversal upon tumor necrosis factor blockade: direct analysis of bone turnover in murine arthritis. Arthritis Res Ther. (2006) 8:R22. doi: 10.1186/ar1872 16507121 PMC1526585

[17] Osta B Benedetti G Miossec P. Classical and paradoxical effects of TNF-alpha on bone homeostasis. Front Immunol. (2014) 5:48. doi: 10.3389/fimmu.2014.00048 24592264 PMC3923157

[18] Wehmeyer C Pap T Buckley CD Naylor AJ. The role of stromal cells in inflammatory bone loss. Clin Exp Immunol. (2017) 189:1–11. doi: 10.1111/cei.12979 28419440 PMC5461090

[19] Kaneki H Guo R Chen D Yao Z Schwarz EM Zhang YE. Tumor necrosis factor promotes Runx2 degradation through up-regulation of Smurf1 and Smurf2 in osteoblasts. J Biol Chem. (2006) 281:4326–33. doi: 10.1074/jbc.M509430200 PMC2647592 16373342

[20] Hess K Ushmorov A Fiedler J Brenner RE Wirth T. TNFalpha promotes osteogenic differentiation of human mesenchymal stem cells by triggering the NF-kappaB signaling pathway. Bone. (2009) 45:367–76. doi: 10.1016/j.bone.2009.04.252 19414075

[21] Huang H Zhao N Xu X Xu Y Li S Zhang J. Dose-specific effects of tumor necrosis factor alpha on osteogenic differentiation of mesenchymal stem cells. Cell Prolif. (2011) 44:420–7. doi: 10.1111/cpr.2011.44.issue-5 PMC6495272 21951285

[22] Briolay A Lencel P Bessueille L Caverzasio J Buchet R Magne D. Autocrine stimulation of osteoblast activity by Wnt5a in response to TNF-alpha in human mesenchymal stem cells. Biochem Biophys Res Commun. (2013) 430:1072–7. doi: 10.1016/j.bbrc.2012.12.036 23266365

[23] Chang J Wang Z Tang E Fan Z McCauley L Franceschi R. Inhibition of osteoblastic bone formation by nuclear factor-kappaB. Nat Med. (2009) 15:682–9. doi: 10.1038/nm.1954 PMC2768554 19448637

[24] Jimenez-Boj E Redlich K Turk B Hanslik-Schnabel B Wanivenhaus A Chott A. Interaction between synovial inflammatory tissue and bone marrow in rheumatoid arthritis. J Immunol. (2005) 175:2579–88. doi: 10.4049/jimmunol.175.4.2579 16081832

[25] Regensburger A Rech J Englbrecht M Finzel S Kraus S Hecht K. A comparative analysis of magnetic resonance imaging and high-resolution peripheral quantitative computed tomography of the hand for the detection of erosion repair in rheumatoid arthritis. Rheumatology. (2015) 54:1573–81. doi: 10.1093/rheumatology/kev031 25832611

[26] Mensah KA Schwarz EM Ritchlin CT. Altered bone remodeling in psoriatic arthritis. Curr Rheumatol Rep. (2008) 10:311–7. doi: 10.1007/s11926-008-0050-5 PMC2656567 18662512

[27] Simon D Kleyer A Faustini F Englbrecht M Haschka J Berlin A. Simultaneous quantification of bone erosions and enthesiophytes in the joints of patients with psoriasis or psoriatic arthritis - effects of age and disease duration. Arthritis Res Ther. (2018) 20:203. doi: 10.1186/s13075-018-1691-z 30170626 PMC6117875

[28] Braun J Sieper J. Ankylosing spondylitis. Lancet. (2007) 369:1379–90. doi: 10.1016/S0140-6736(07)60635-7 17448825

[29] Hugle T Geurts J. What drives osteoarthritis?-synovial versus subchondral bone pathology. Rheumatology. (2017) 56:1461–71. doi: 10.1093/rheumatology/kew389 28003493

[30] Atkins RM Langkamer VG Perry MJ Elson CJ Collins CM. Bone-membrane interface in aseptic loosening of total joint arthroplasties. J Arthroplasty. (1997) 12:461–4. doi: 10.1016/S0883-5403(97)90203-5 9195323

[31] Jeffcoat MK Williams RC Holman BL English R Goldhaber P. Detection of active alveolar bone destruction in human periodontal disease by analysis of radiopharmaceutical uptake after a single injection of 99m-Tc-methylene diphosphonate. J periodontal Res. (1986) 21:677–84. doi: 10.1111/j.1600-0765.1986.tb01505.x 2948001

[32] El-Shanti HI Ferguson PJ. Chronic recurrent multifocal osteomyelitis: a concise review and genetic update. Clin Orthop Relat Res. (2007) 462:11–9. doi: 10.1097/BLO.0b013e3180986d73 17496555

[33] Vannet NB Williams HL Healy B Morgan-Jones R. Sclerosing osteomyelitis of Garre: management of femoral pain by intramedullary nailing. BMJ Case Rep. (2014) 2014:1–8. doi: 10.1136/bcr-2014-206533 PMC4275760 25538212

[34] Johnson RW Suva LJ. Hallmarks of bone metastasis. Calcif Tissue Int. (2018) 102:141–51. doi: 10.1007/s00223-017-0362-4 PMC5807131 29138883

[35] Gortz B Hayer S Redlich K Zwerina J Tohidast-Akrad M Tuerk B. Arthritis induces lymphocytic bone marrow inflammation and endosteal bone formation. J Bone Miner Res. (2004) 19:990–8. doi: 10.1359/JBMR.040205 15125796

[36] Yoshii T Magara S Miyai D Nishimura H Kuroki E Furudoi S. Local levels of interleukin-1beta, -4, -6 and tumor necrosis factor alpha in an experimental model of murine osteomyelitis due to staphylococcus aureus. Cytokine. (2002) 19:59–65. doi: 10.1006/cyto.2002.1039 12182840

[37] Moverare-Skrtic S Henning P Liu X Nagano K Saito H Borjesson AE. Osteoblast-derived WNT16 represses osteoclastogenesis and prevents cortical bone fragility fractures. Nat Med. (2014) 20:1279–88. doi: 10.1038/nm.3654 PMC4392888 25306233

[38] Kassem A Henning P Lundberg P Souza PP Lindholm C Lerner UH. *Porphyromonas gingivalis* Stimulates Bone Resorption by Enhancing RANKL (Receptor Activator of NF-kappaB Ligand) through Activation of Toll-like Receptor 2 in Osteoblasts. J Biol Chem. (2015) 290:20147–58. doi: 10.1074/jbc.M115.655787 PMC4536425 26085099

[39] Boyce BF Aufdemorte TB Garrett IR Yates AJ Mundy GR. Effects of interleukin-1 on bone turnover in normal mice. Endocrinology. (1989) 125:1142–50. doi: 10.1210/endo-125-3-1142 2788075

[40] Windahl SH Vidal O Andersson G Gustafsson JA Ohlsson C. Increased cortical bone mineral content but unchanged trabecular bone mineral density in female ERbeta(-/-) mice. J Clin Invest. (1999) 104:895–901. doi: 10.1172/JCI6730 10510330 PMC408552

[41] Vidal O Lindberg MK Hollberg K Baylink DJ Andersson G Lubahn DB. Estrogen receptor specificity in the regulation of skeletal growth and maturation in male mice. Proc Natl Acad Sci USA. (2000) 97:5474–9. doi: 10.1073/pnas.97.10.5474 PMC25853 10805804

[42] Nethander M Moverare-Skrtic S Kampe A Coward E Reimann E Grahnemo L. An atlas of genetic determinants of forearm fracture. Nat Genet. (2023) 55:1820–30. doi: 10.1038/s41588-023-01527-3 PMC10632131 37919453

[43] Martin TJ Sims NA. RANKL/OPG; Critical role in bone physiology. Rev endocrine Metab Disord. (2015) 16:131–9. doi: 10.1007/s11154-014-9308-6 25557611

[44] Cohen SB Dore RK Lane NE Ory PA Peterfy CG Sharp JT. Denosumab treatment effects on structural damage, bone mineral density, and bone turnover in rheumatoid arthritis: a twelve-month, multicenter, randomized, double-blind, placebo-controlled, phase II clinical trial. Arthritis rheumatism. (2008) 58:1299–309. doi: 10.1002/art.23417 18438830

[45] Takeuchi T Tanaka Y Ishiguro N Yamanaka H Yoneda T Ohira T. Effect of denosumab on Japanese patients with rheumatoid arthritis: a dose-response study of AMG 162 (Denosumab) in patients with RheumatoId arthritis on methotrexate to Validate inhibitory effect on bone Erosion (DRIVE)-a 12-month, multicentre, randomised, double-blind, placebo-controlled, phase II clinical trial. Ann rheumatic Dis. (2016) 75:983–90. doi: 10.1136/annrheumdis-2015-208052 PMC4893103 26585988

[46] Russell RG. Bisphosphonates: the first 40 years. Bone. (2011) 49:2–19. doi: 10.1016/j.bone.2011.04.022 21555003

[47] Baron R Kneissel M. WNT signaling in bone homeostasis and disease: from human mutations to treatments. Nat Med. (2013) 19:179–92. doi: 10.1038/nm.3074 23389618

[48] Lerner UH Ohlsson C. The WNT system: background and its role in bone. J Int Med. (2015) 277:630–49. doi: 10.1111/joim.12368 25845559

[49] Sims NA Martin TJ. Coupling signals between the osteoclast and osteoblast: How are messages transmitted between these temporary visitors to the bone surface? Front Endocrinol. (2015) 6:41. doi: 10.3389/fendo.2015.00041 PMC4371744 25852649

[50] Gruneboom A Hawwari I Weidner D Culemann S Muller S Henneberg S. A network of trans-cortical capillaries as mainstay for blood circulation in long bones. Nat Metab. (2019) 1:236–50. doi: 10.1038/s42255-018-0016-5 PMC6795552 31620676

[51] Herisson F Frodermann V Courties G Rohde D Sun Y Vandoorne K. Direct vascular channels connect skull bone marrow and the brain surface enabling myeloid cell migration. Nat Neurosci. (2018) 21:1209–17. doi: 10.1038/s41593-018-0213-2 PMC6148759 30150661

[52] Balemans W Ebeling M Patel N Van Hul E Olson P Dioszegi M. Increased bone density in sclerosteosis is due to the deficiency of a novel secreted protein (SOST). Hum Mol Genet. (2001) 10:537–43. doi: 10.1093/hmg/10.5.537 11181578

[53] Balemans W Patel N Ebeling M Van Hul E Wuyts W Lacza C. Identification of a 52 kb deletion downstream of the SOST gene in patients with van Buchem disease. J Med Genet. (2002) 39:91–7. doi: 10.1136/jmg.39.2.91 PMC1735035 11836356

[54] Cosman F Crittenden DB Adachi JD Binkley N Czerwinski E Ferrari S. Romosozumab treatment in postmenopausal women with osteoporosis. N Engl J Med. (2016) 375:1532–43. doi: 10.1056/NEJMoa1607948 27641143

[55] Saag KG Petersen J Brandi ML Karaplis AC Lorentzon M Thomas T. Romosozumab or alendronate for fracture prevention in women with osteoporosis. N Engl J Med. (2017) 377:1417–27. doi: 10.1056/NEJMoa1708322 28892457

[56] Funck-Brentano T Nilsson KH Brommage R Henning P Lerner UH Koskela A. Porcupine inhibitors impair trabecular and cortical bone mass and strength in mice. J Endocrinol. (2018) 238:13–23. doi: 10.1530/JOE-18-0153 29720540 PMC5987170

[57] Lawson LY Brodt MD Migotsky N Chermside-Scabbo CJ Palaniappan R Silva MJ. Osteoblast-specific wnt secretion is required for skeletal homeostasis and loading-induced bone formation in adult mice. J Bone Miner Res. (2022) 37:108–20. doi: 10.1002/jbmr.4445 PMC8770559 34542191

[58] Hu H Hilton MJ Tu X Yu K Ornitz DM Long F. Sequential roles of Hedgehog and Wnt signaling in osteoblast development. Development. (2005) 132:49–60. doi: 10.1242/dev.01564 15576404

[59] Tu X Joeng KS Nakayama KI Nakayama K Rajagopal J Carroll TJ. Noncanonical Wnt signaling through G protein-linked PKCdelta activation promotes bone formation. Dev Cell. (2007) 12:113–27. doi: 10.1016/j.devcel.2006.11.003 PMC1861818 17199045

[60] Chen J Tu X Esen E Joeng KS Lin C Arbeit JM. WNT7B promotes bone formation in part through mTORC1. PLoS Genet. (2014) 10:e1004145. doi: 10.1371/journal.pgen.1004145 24497849 PMC3907335

[61] Feng B Pei J Gu S. Wnt7b: Is it an important factor in the bone formation process after calvarial damage? J Clin Med. (2023) 12:1–11. doi: 10.3390/jcm12030800 PMC9917507 36769446

[62] Yu F Wu F Li F Liao X Wang Y Li X. Wnt7b-induced Sox11 functions enhance self-renewal and osteogenic commitment of bone marrow mesenchymal stem cells. Stem Cells. (2020) 38:1020–33. doi: 10.1002/stem.3192 32346881

[63] Wu F Li B Hu X Yu F Shi Y Ye L. Wnt7b inhibits osteoclastogenesis *via* AKT activation and glucose metabolic rewiring. Front Cell Dev Biol. (2021) 9:771336. doi: 10.3389/fcell.2021.771336 34881243 PMC8645835

[64] Tsukamoto S Kuratani M Tanaka S Jimi E Oda H Katagiri T. Wnt7b expressed by hypertrophic chondrocytes is a stimulatory factor for endochondral ossification that is regulated by Smad4 activity. Development. (2023) 150:1–9. doi: 10.1242/dev.201734 37539462

[65] Liu LJ Lv Z Xue X Xing ZY Zhu F. Canonical WNT signaling activated by WNT7B contributes to L-HBs-mediated sorafenib resistance in hepatocellular carcinoma by inhibiting mitophagy. Cancers. (2022) 14:1–17. doi: 10.3390/cancers14235781 PMC9741164 36497264

