# Supplementary figures and images for: Toll-like receptor-2 induced inflammation causes local bone formation and activates canonical Wnt signaling
Source: Front Immunol. 2024 Apr 5;15:1383113. doi: 10.3389/fimmu.2024.1383113 (PMC11026618; doi:10.3389/fimmu.2024.1383113)

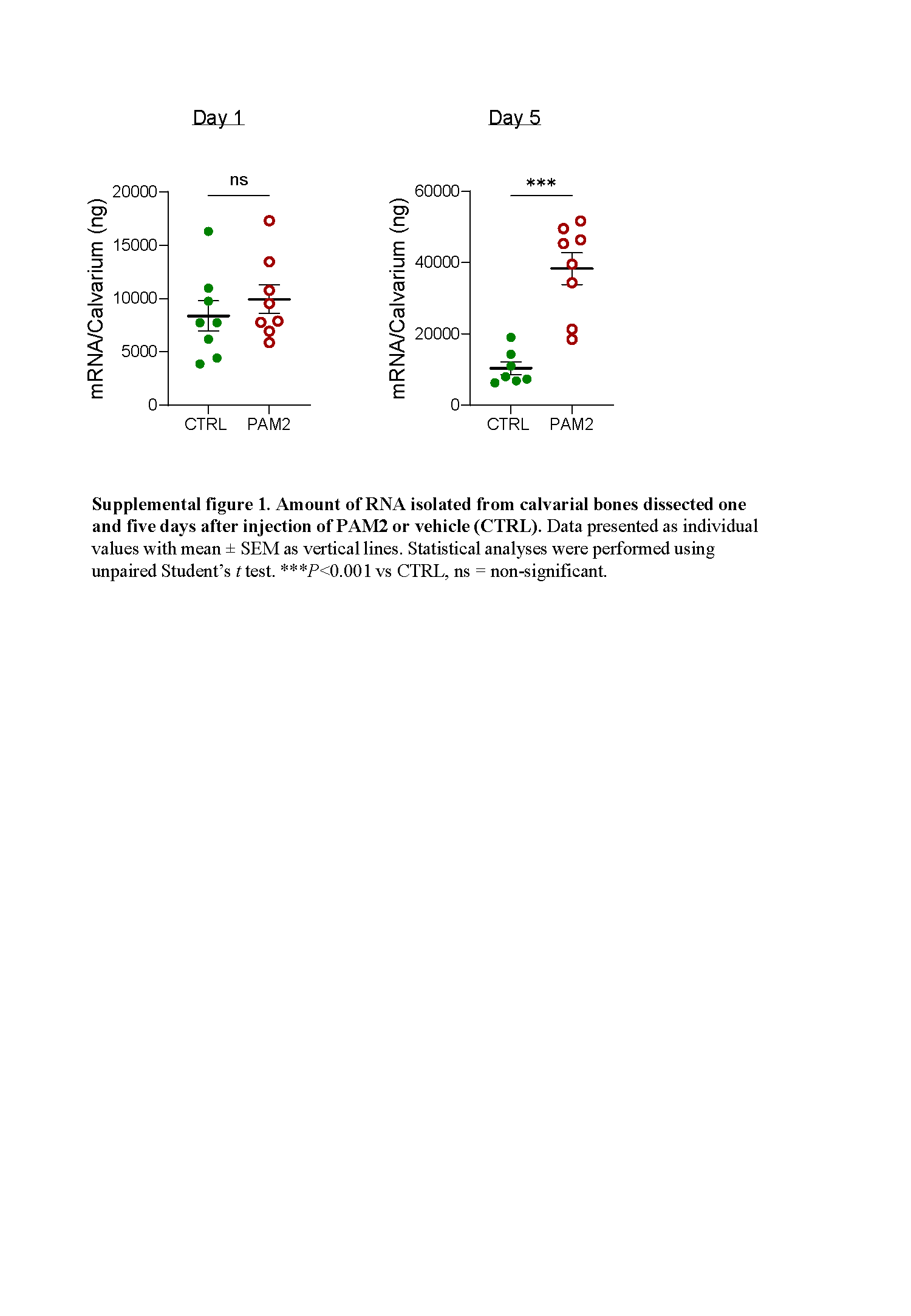

Supplement: Supplementary file 1 [file Image_1.tif]

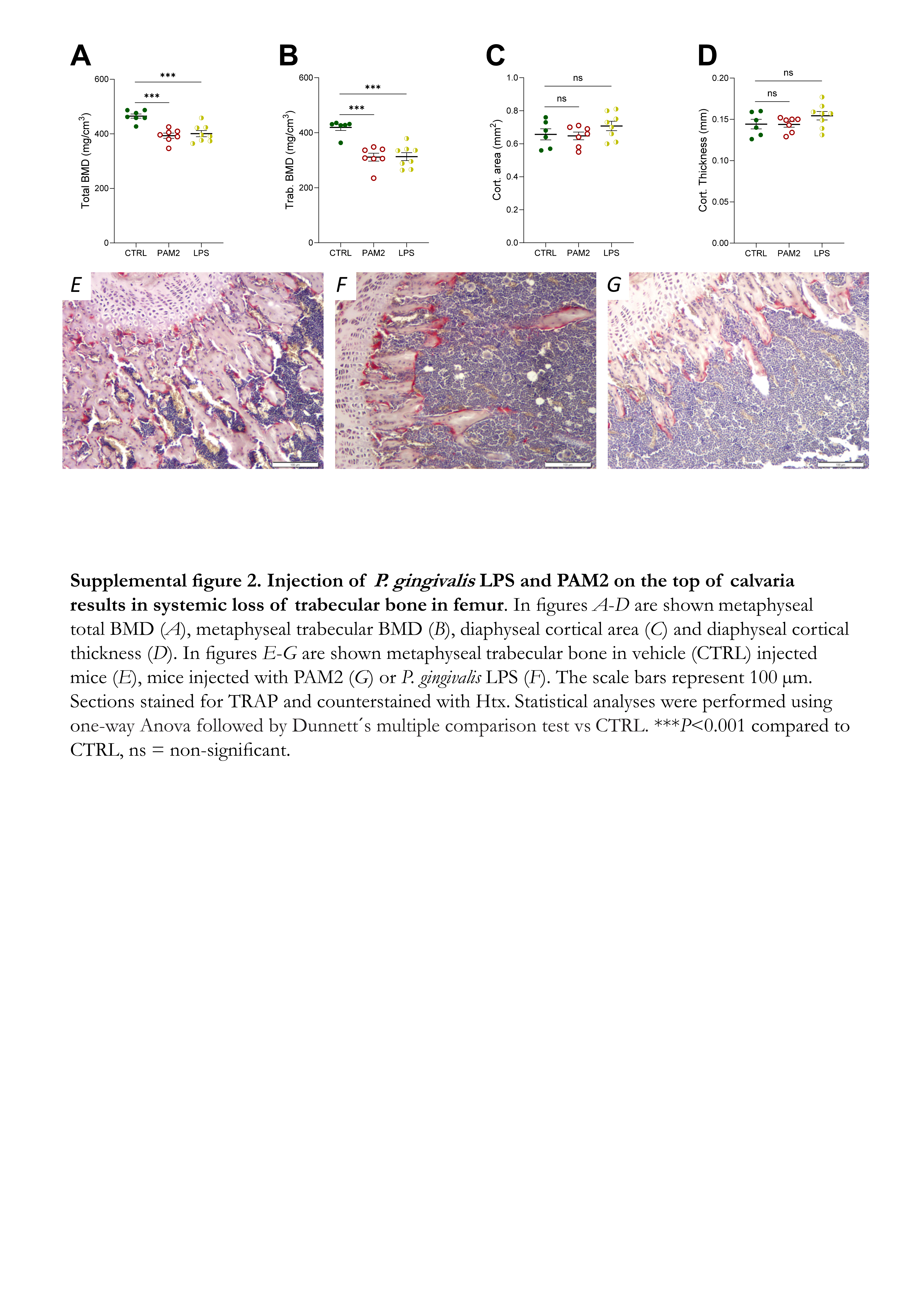

Supplement: Supplementary file 2 [file Image_2.tiff]

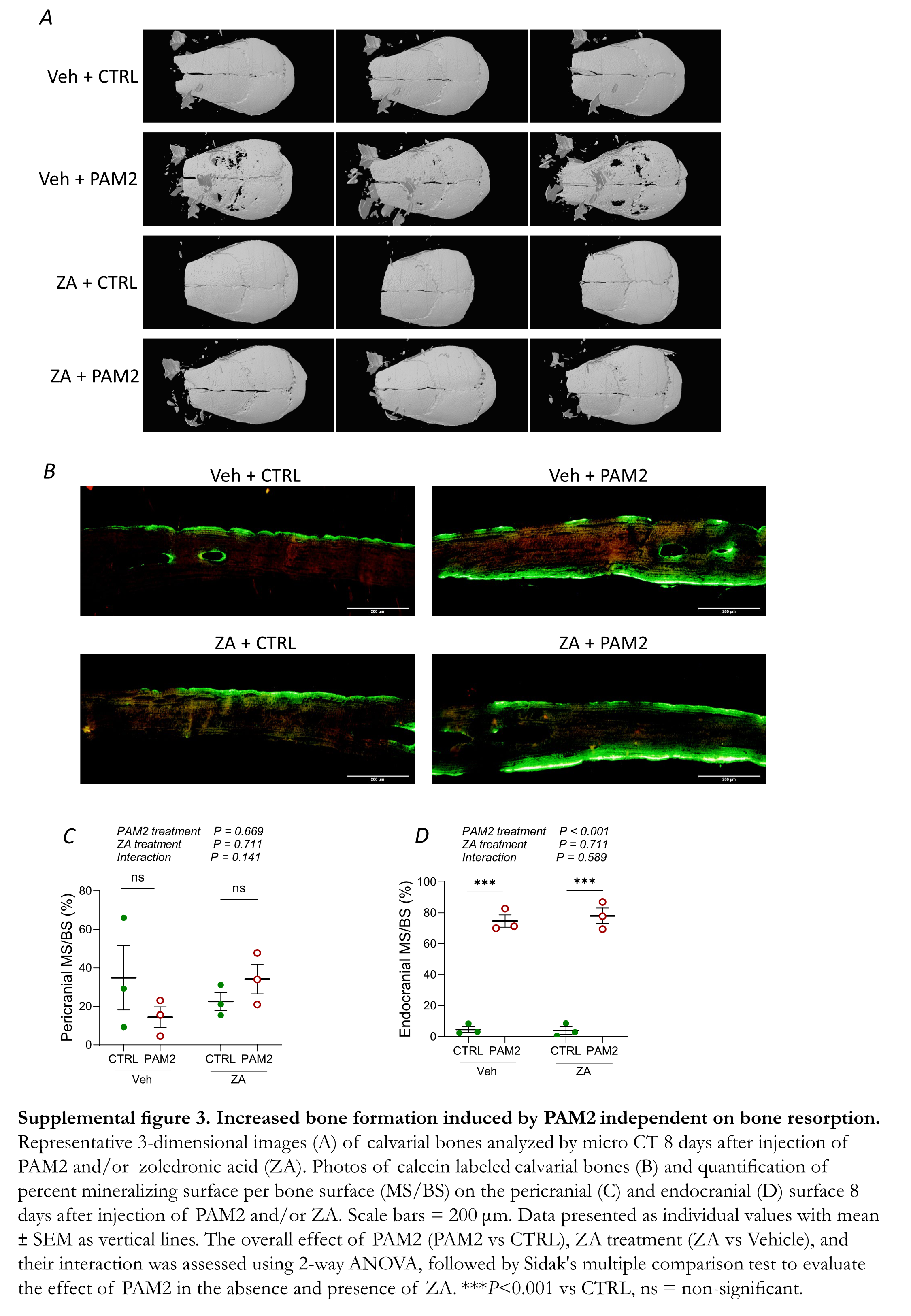

Supplement: Supplementary file 3 [file Image_3.tiff]

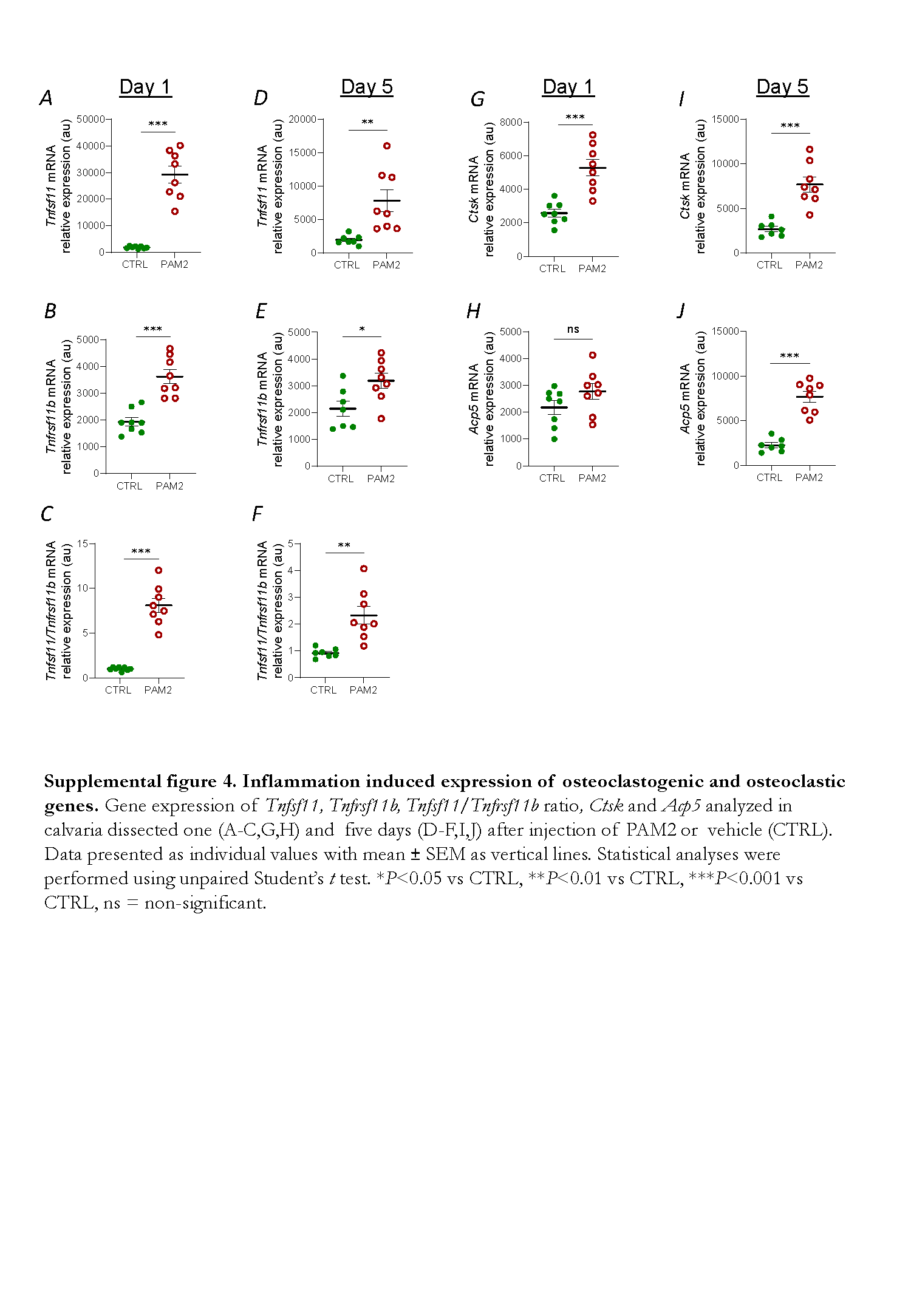

Supplement: Supplementary file 4 [file Image_4.tif]

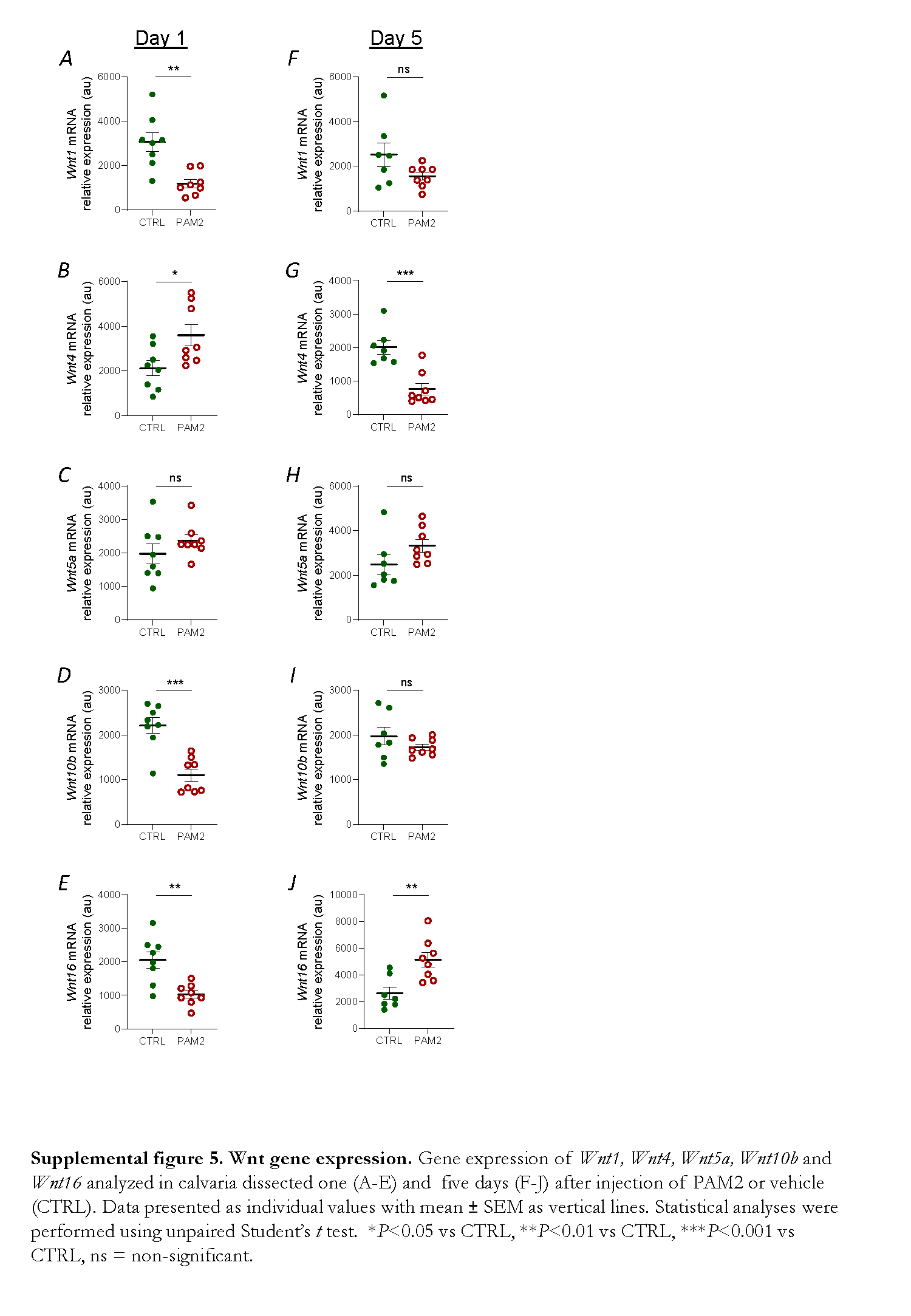

Supplement: Supplementary file 5 [file Image_5.tif]
